# The Oviposition Inhibitory Neuron is a Potential Hub of Multi-Circuit Integration in the *Drosophila* Brain

**DOI:** 10.1523/ENEURO.0123-25.2025

**Published:** 2025-09-10

**Authors:** Rhessa A. Weber-Langstaff, Pranjal Srivastava, Alexander B. Kunin, Gabrielle J. Gutierrez

**Affiliations:** ^1^Department of Neuroscience and Behavior, Barnard College, New York, New York 10027; ^2^Department of Computer Science, Columbia University, New York, New York 10027; ^3^Department of Mathematics, Creighton University Omaha, Nebraska 68178

**Keywords:** community detection, connectome, modularity, network analysis, network neuroscience, neuronal circuits, neuropils, superior protocerebrum

## Abstract

Understanding how neural circuits integrate sensory and state information to support context-dependent behavior is a central challenge in neuroscience. Oviposition is a complex process during which a fruit fly integrates context and sensory information to choose an optimal location to lay her eggs. The circuit that controls oviposition is known, but how the oviposition circuit integrates multiple sensory modalities and internal states is not. Using the Hemibrain connectome, we identified the oviposition inhibitory neuron (oviIN) as a key hub in the oviposition circuit and analyzed its inputs to uncover potential parallel pathways that may be responsible for computations related to sensory integration and decision-making. We applied a network analysis to the subconnectome of inputs to the oviIN to identify clusters of interconnected neurons—many of which are uncharacterized cell types. Our findings indicate that the inputs to oviIN form multiple parallel pathways through the unstructured neuropils of the superior protocerebrum, a region implicated in context-dependent processing.

## Significance Statement

The recent advent of the *Drosophila* connectome enables researchers to probe the connectivity of uncharacterized cell types in the parts of the fruit fly brain that are responsible for cognitive-level computations. Our study analyzed the connectivity of the oviposition circuit which controls a complex behavior that depends on sensory and context integration and decision-making computations. Using graph-theoretic and computational methods, we found that the sole inhibitory neuron in the circuit is a hub that integrates information from multiple clusters of uncharacterized neurons with potentially novel functions. Our work presents a new and timely perspective by demonstrating how new targets for study can be identified from the vast trove of uncharacterized neurons and cell types in the connectome.

## Introduction

Decision-making is a widely accepted facet of cognition (Gold and Shadlen, 2007; Shadlen and Kiani, 2013). Although cognitive abilities are not often attributed to *Drosophila*, these animals are known to evaluate their available options to make decisions about what to eat (Burke and Waddell, 2011; Fujita and Tanimura, 2011; Itskov and Ribeiro, 2013; Yu et al., 2021; Deere and Devineni, 2022), and where to lay eggs (Yang et al., 2008; Joseph et al., 2009; Azanchi et al., 2013; Cury et al., 2019; Zhang et al., 2020; Churchill et al., 2021; Vijayan et al., 2022). After mating, the female fly assesses a variety of factors such as the firmness (Zhang et al., 2020), taste (Yang et al., 2008; Joseph et al., 2009; Vijayan et al., 2022), and spatial attributes of a substrate (Schwartz et al., 2012), before deciding whether to deposit an egg. While sampling and evaluating multiple sensory inputs, the fly computes a relative value for each encountered substrate (Yang et al., 2008; Azanchi et al., 2013; Yang et al., 2015; Vijayan et al., 2023). The fly considers social contexts and physical environment to optimize survival of the egg (Rockwell and Grossfield, 1978; Shelly, 1999; Schwartz et al., 2012; Bailly et al., 2023). Identifying the circuits responsible for such cognitive-level processing within the vast network of the *Drosophila* brain presents a significant challenge (O’Leary and Marder, 2014). Partial and full-brain connectomes provide the most comprehensive map of the connectivity in the *Drosophila* brain currently available (Scheffer et al., 2020; Dorkenwald et al., 2024; Schlegel et al., 2024), although there is some variability between connectomes (Lin et al., 2024).

**Figure 1.**
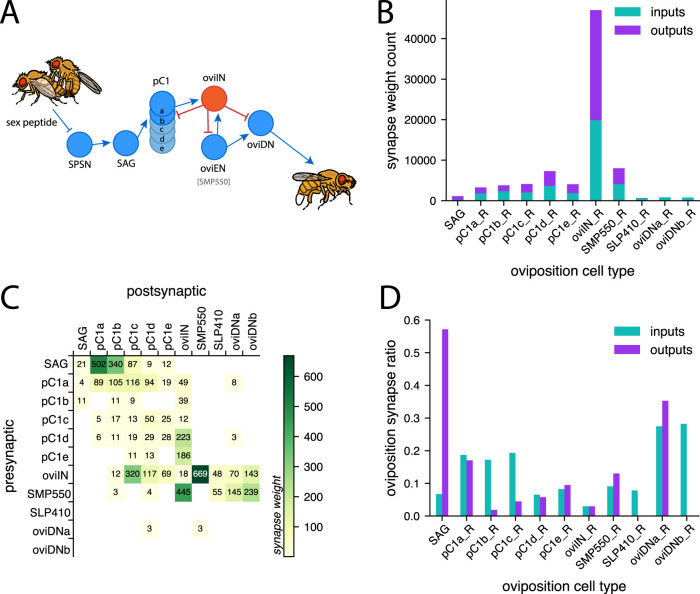
The connectivity profile for the oviposition circuit in the Hemibrain connectome. ***A***, Schematic circuit diagram of the oviposition circuit from Wang et al. (2020) that conveys mating status to the oviposition descending neurons (oviDN) which command the oviposition motor sequence. The oviIN (red) is an inhibitory neuron in this circuit. Fly illustrations are reused and adapted from DataBase Center for Life Science (DBCLS), https://doi.org/10.7875/togopic.2022.325. ***B***, Total synapse counts for the oviposition cell types in the Hemibrain connectome. Counts are based on the number of presynaptic (inputs; teal) and postsynaptic (outputs; purple) synapse connections made by each cell type instance shown (connection weights <3 are excluded from the tally). Only right instances of the oviposition cell types are included because left instances are often truncated. The mean counts are presented for the SAG and SLP410_R because they each have two instances. The SMP550 are presumed to be the oviEN, and the SLP410 are believed to be an additional pair of the oviDNa subtype (Nojima et al., 2021). See Extended Data Figure 1-1 for extent of overlap among presynaptic partners. ***C***, Connectivity matrix for the oviposition cell types. Connection weights <3 are excluded. Synapse counts are the aggregated synaptic weights from all neurons of one cell type to all neurons of another cell type (combining right and left instances). ***D***, The proportion of synapses that are made with other oviposition neurons. The inputs ratio (teal) for a given cell type is the proportion of synapses received from presynaptic oviposition partners relative to synapses from all presynaptic partners. The outputs ratio (purple) is the proportion of synapses to other oviposition neurons relative to synapses to all postsynaptic partners. Connection weights <3 are excluded from this analysis.

The circuit responsible for commanding oviposition and the cell types involved are known (Wang et al., 2020). Within each hemisphere, a set of oviposition descending neurons (oviDN) are directly downstream of a single oviposition excitatory neuron (oviEN) and oviposition inhibitory neuron (oviIN; Fig. 1*A*). Mating status is conveyed to the oviDN via the oviIN by way of a subset of pC1 neurons. The pC1 cluster of neurons is a sexually dimorphic cell type that promotes persistent aggressive behaviors (Deutsch et al., 2020; Schretter et al., 2020) and sexual receptivity (Zhou et al., 2014). Following mating, sex peptide inhibits the sex peptide sensory neurons (SPSN) and sex peptide abdominal ganglia (SAG) neurons (Feng et al., 2014; Wang et al., 2020; Laturney et al., 2023). The pC1a and pC1b neurons downstream of SPSN and SAG are silenced by the reduced input which in turn silences the oviIN. The result is the disinhibition of the oviDN which elicit the oviposition motor action (Wang et al., 2020). While this neural pathway is responsible for encoding and conveying mating status (Yang et al., 2009; Wang et al., 2020), it does not account for other computations that influence oviposition, such as the evaluations of substrate firmness and nutrient content, which depend on sensory inputs. The neural correlates of these computations are not yet well-defined, but multiple circuits may converge onto the oviposition circuit to influence decision-making and eventually egg-laying behavior.

10.1523/ENEURO.0123-25.2025.f1-1 Figure 1-1 The overlap in unique presynaptic partners to oviIN_R (pink), SMP550_R (oviEN; green), and the combined group of right oviDN subtypes (oviDNa_R, oviDNb_R, and both instances of SLP410_R; purple). There are 14 unique neurons that are presynaptic to all 3 cell types. Download Figure 1-1, TIF file.

In our present study, we begin an exploration of the understudied areas that influence oviposition decision-making by focusing our analyses on the inputs to one central player in the oviposition circuit. Our initial analyses revealed that the oviIN is a highly interconnected neuron in the oviposition circuit and in the brain. We find that the inputs to oviIN predominantly come from the superior neuropils of the protocerebrum (SNP) which include the superior lateral protocerebrum (SLP), the superior intermediate protocerebrum (SIP), and the superior medial protocerebrum (SMP; Ito et al., 2014). These structures have been known to support high-level processing of taste (Kim et al., 2017), water-seeking behavior (Landayan et al., 2021) that depends on the complex interactions between hunger and thirst (Jourjine et al., 2016; González Segarra et al., 2023) , locomotor control (Marquis and Wilson, 2022), and persistent internal states in female flies (Deutsch et al., 2020). Unlike the physical ring attractor within the central complex (CX) or the clearly compartmentalized mushroom body (MB), the SNP do not have a structure that reveals their function, and are described as a “diffuse” set of neuropils (Ito et al., 2014). We employed a graph-theoretic approach to identify community structure among the inputs to the oviIN. Our analysis revealed clusters of neurons that preferentially target the oviIN from diffuse regions of the central brain. These clusters make anatomically organized synapses on the branches of the oviIN. Ultimately, determining the organization of oviIN’s inputs will facilitate the discovery of novel circuits involved in high-level processing.

## Methods and Materials

### Accessing and working with the Hemibrain connectome data

**Figure 2.**
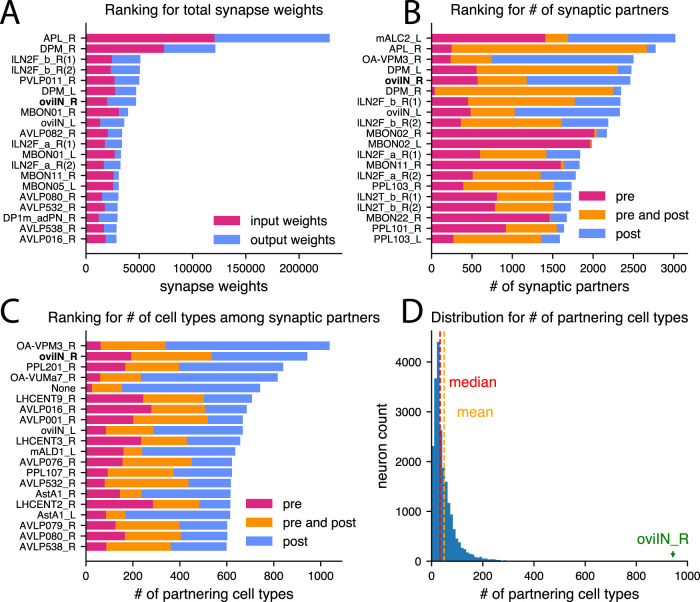
The oviIN is an outlier in the Hemibrain. ***A***, Out of the 21,739 traced, non-cropped neurons in the Hemibrain, the top 20 neurons with the highest total synaptic connections with traced neurons are ranked in descending order and labeled according to their Hemibrain cell type and whether it is a right (R) or left (L) instance (the number in parentheses indicates whether there are multiple instances appearing in the ranking). The right oviIN (oviIN_R) ranks seventh with 47,044 synaptic connections. Input weights (magenta) are counts of postsynaptic sites on the indicated neuron that are paired with presynaptic sites from other neurons. Output weights (blue) are counts of postsynaptic sites that are paired with the presynaptic sites on the indicated neuron. ***B***, The top 20 traced, non-cropped neurons in the Hemibrain with the greatest quantity of unique neuron partners are ranked in descending order and labeled as in (***A***). Counts of partner neurons that are exclusively presynaptic are shown in magenta, exclusively postsynaptic partners are shown in blue, and counts of neurons that are both presynaptic and postsynaptic to the indicated neuron are shown in orange. Partners making connection weights <3 were excluded. The oviIN_R ranks 5th with 2,460 total partners (1,180 presynaptic and 1,890 postsynaptic neurons, of which 610 neurons are both presynaptic and postsynaptic). ***C***, The top 20 traced, non-cropped neurons in the Hemibrain with the greatest quantity of distinct cell types among their direct synaptic partners are ranked in descending order and labeled as in (***A***). Counts of cell types that are exclusively presynaptic are shown in magenta, exclusively postsynaptic cell types are shown in blue, and counts of cell types that are both presynaptic and postsynaptic to the indicated neuron are shown in orange. Connection weights <3 between pairs of neurons were excluded. The oviIN_R ranks second with 943 total cell types among its partners (536 presynaptic and 750 postsynaptic cell types, of which 343 cell types are both presynaptic and postsynaptic). ***D***, Histogram showing the distribution of the number of cell types among synaptic partners for all neurons in the Hemibrain. The median neuron partners with 33 cell types while oviIN_R makes direct connections with 943 cell types.

All analyses presented were performed using the publicly available Hemibrain connectome data released by Janelia (Scheffer et al., 2020). The data are from an EM reconstruction of the right hemisphere and partial left hemisphere of a single female fly brain. Morphological data for the 3D reconstruction was accessed, along with tabular data of the neuron annotations and connectivity, using the Neuprint-python package (https://github.com/connectome-neuprint/neuprint-python). The data accessed via Neuprint is Hemibrain version 1.2.1. For the brain-wide rankings (Fig. 2), we relied on the most recent table of traced neurons made available by Janelia (https://storage.cloud.google.com/hemibrain/v1.2/exported-traced-adjacencies-v1.2.tar.gz) which is version 1.2, and which contains only neurons that are traced and uncropped.

Every contiguous object in the Hemibrain is assigned a unique bodyId, and many of these are also assigned a cell type. Janelia annotated cell types based on their morphologies and connectivity patterns. There are some traced, non-cropped neurons that are not assigned a cell type. These are labeled as “None” and were excluded from certain analyses wherever indicated. Janelia also annotates neurons with an instance label that can be used to differentiate between right and left instances of bilateral cell types. For example, there are two neurons in the Hemibrain of the oviIN cell type: a right and a left instance that are denoted as oviIN_R and oviIN_L, respectively. Our analyses largely focused on the right oviIN.

Neurons in the Hemibrain that match with neurons that have been empirically characterized in the literature are typically annotated with their known name, thus we denote these as characterized cell types. Neurons that have not been characterized are often given a cell type name that begins with the abbreviation for the neuropil where most of its synaptic contacts are located followed by a three-digit number where the numbering sequence begins with 001. Neurons such as these may have been previously characterized in the literature but have not been matched in the Hemibrain. One such example is SMP550 which is presumed by Nojima et al. (2021) to be the oviEN that Wang et al. (2020) identified in the FAFB volume, but the Hemibrain has not updated its cell type label to reflect that proposition. Except for this particular case, we denote generically labeled and unnamed cell types (“None”) as uncharacterized.

*Drosophila* synapses are polyadic, meaning that multiple postsynaptic densities can be associated with a single presynaptic T-bar. The Hemibrain provides counts of the postsynaptic density sites as well as counts of the presynaptic sites on the bodies of anything that is given a bodyId, as well as locations in 3D space of those sites. Additionally, the Hemibrain provides data for the synaptic connections among neurons in the 3D reconstruction. These are given as synaptic weights where the weight is the count of postsynaptic sites within the synapses that connect two neurons. A connection weight between a pair of unique neurons that is <3 can be considered a potentially erroneous connection (Scheffer et al., 2020). We implement a connection strength threshold of 3 for several of the analyses in our study, wherever indicated.

#### Primacy of inputs to oviIN

A neuron or cell type is considered a primary input to oviIN if it has oviIN as its strongest output. Primacy is determined on a by-cell type basis by querying the synaptic outputs of all the neurons of a given cell type, summing those weights by postsynaptic cell type, and putting those aggregated weights in descending order. The position of the oviIN type in the list determines the primacy of oviIN as an output. If oviIN is in the first position of outputs from a neuron, that neuron is a primary input to oviIN (Fig. 4*B*).

#### Parsing neuropil data

When fetching adjacencies between neurons or cell types in the Hemibrain, the data indicates the neuropil, or region of interest (ROI), in which the connection is located. For consistency and clarity, we merged right and left ROIs and relabel primary ROIs with their corresponding supercategory according to Ito et al. (2014; Extended Data Fig. 5-1).

For bar chart visualizations in which the data were restricted to synapses made with oviIN (Extended Data Fig. 4-8), the synapse weights between oviIN_R and its presynaptic partners were queried by ROI. For each module, the synapse weights from module neurons onto oviIN_R were summed within each neuropil supercategory, thus providing the total synapse weights onto oviIN_R made within each supercategory by the neurons in a given module. For the bar chart visualization in Figure 5*A*, all presynaptic connections to presynaptic partners of oviIN_R were queried. For each module, the synapse weights from any neuron in the brain onto a presynaptic partner of oviIN_R were summed within each supercategory, thus providing the total synapse weights within each supercategory onto oviIN_R’s presynaptic partners within a given module.

### RenEEL method for computing generalized modularity

We used reduced network extremal ensemble learning (RenEEL, Guo et al., 2019), a machine learning based approach for network clustering, to identify the partition C which maximizes the generalized modularity density (Eq. 1; Guo et al., 2023). RenEEL first creates an ensemble of candidate partitions using a fast heuristic. The ensemble is iteratively updated, alternating steps of reducing and replacing. In the reducing step, each community identified by all partitions in the ensemble is combined to a single node. In the replacing step, the fast heuristic is used again on the reduced network. If the result matches one of the previous partitions, or has a lower score than any of the ensemble partitions, the ensemble size is reduced by one. Otherwise, the result replaces the ensemble partition with the lowest modularity score. This process repeats until only one partition remains in the ensemble, representing a consensus best partition. We used an implementation that has previously been shown to produce fast and accurate results for networks of the size and density we consider (Guo et al., 2019, 2023; Kunin et al., 2023). The source code is available on Github: https://github.com/prameshsingh/generalized-modularity-density.

The set of neurons presynaptic to oviIN_R and the synaptic connections among them were converted into an undirected, weighted graph. The weight of each edge was the total synaptic weight between the two neurons in both directions. The community structure of the network was identified with the partition $C=\{C_{1},C_{2},\ldots \}$ maximizing the generalized modularity density score (Guo et al., 2023):

$$Q_{g}=\sum_{C\in C}\left(\frac{m_{C}}{m}-(\frac{K_{C}}{2m}{)}^{2}\right)\rho_{C}^{\chi }.$$

The sum is taken over communities *C*; *m* is the total number of edges in the network, *m*_*C*_ is the total weight of edges in community *C*, *K*_*C*_ is the weight-degree sum of nodes in *C* (the sum of the weights of edges incident to each node in *C*), and *ρ*_*C*_ is the relative density of connections in *C*, defined by:

$$\rho_{C}=\frac{2m_{C}}{n_{C}(n_{C}-1)},$$

where *n*_*C*_ is the number of nodes in community *C*. The exponent *χ* controls the resolution of the clustering. At *χ* = 0, *Q*_*g*_ equals classic modularity density (Newman, 2006), with *χ* > 0 resolving smaller, more densely connected communities (Guo et al., 2023).

In our study, RenEEL was applied to the subconnectome of oviIN_R inputs which consists of all the presynaptic partners to oviIN_R and the connections among them. Only traced, non-cropped neurons were included in this subconnectome in keeping with the analyses done by Kunin et al. (2023). To avoid isolating any nodes that could be potentially informative to the clustering, no synaptic weight threshold was applied to the inputs to oviIN_R nor to the connections among those inputs. Thus, the subconnectome was overly inclusive and it contained 1,832 nodes as well as all connections with non-zero weight. The results presented are from a representative run (using *χ* = 0) that resulted in a single set of partitions, C. RenEEL was subsequently run on the same subconnectome 30 times with different random seeds to determine that partitions were largely consistent between runs (mean Jaccard similarity: 0.89). The weak inputs to oviIN drove most of the variability in the results while the primary strong inputs to oviIN were more consistently partitioned. When analyzing and making comparisons to the modularity of the full Hemibrain, RenEEL results from Kunin et al. (2023) were used.

### Analyses and visualizations

#### Code accessibility

All analyses and visualizations were done in Python using custom-written scripts along with various published and open-source packages. Our analysis code is publicly available on GitHub: https://github.com/Gutierrez-lab/oviIN-inputs. We used Mac OS computers for most work, and a high performance computing cluster for running the RenEEL algorithm on the connectome data.

#### Joint clustering plots

We compared the modularity results for the oviIN_R inputs subconnectome clustering and the full Hemibrain connectome clustering. We computed the proportion of nodes from a module in one clustering that are partitioned into a module from the other clustering. The width and height of the boxes in Figure 5*B* represent the joint counts of neurons within two modules over the total counts within a module of the respective dataset. If $N_{H_{i}}$ is the number of nodes in module *i* of the full Hemibrain clustering, and $N_{O_{j}}$ is the number of nodes in module *j* of the oviIN_R input clustering, and the number of nodes jointly appearing in module *i* of the full Hemibrain clustering and in module *j* of the oviIN_R input clustering is $N_{H_{i}\cap O_{j}}$, then the width of any box is $N_{H_{i}\cap O_{j}}/N_{O_{j}}$ and the height of any box is $N_{H_{i}\cap O_{j}}/N_{H_{i}}$.

#### Jaccard similarity

To evaluate the similarity between different neuron clusterings A and B, we compute the Jaccard similarity. For a set of neurons {1, 2, 3, …*N*}, Jaccard Similarity considers all pairs of neurons in the set and is defined as:

$$J(A,B)=\frac{\sum_{i=1}^{N}\sum_{\;j=i+1}^{N}1\{C_{i}^{A}=C_{j}^{A}\;\text{and}\;C_{i}^{B}=C_{j}^{B}\}}{\sum_{i=1}^{N}\sum_{\;j=i+1}^{N}1\{C_{i}^{A}=C_{j}^{A}\;\text{or}\;C_{i}^{B}=C_{j}^{B}\}},$$

where *N* is the total number of neurons in the set, $C_{i}^{A}$ and $C_{i}^{B}$ are the cluster assignments of neuron *i* in clusterings A and B, respectively. The indicator function **1**{ · } equals 1 if the condition inside is true and 0 otherwise.

The Jaccard similarity ranges from 0 to 1. A value of 0 indicates no overlap between the clusterings, while a value of 1 indicates perfect overlap. Intermediate values indicate that some pairs of neurons are clustered together for both clusterings. In our study, we applied this method to compare the clustering of the oviIN_R inputs with the clustering of the entire Hemibrain for different resolutions of RenEEL algorithm.

#### Input similarity

Input similarity was computed as the cosine distance between the vectors representing the inputs to each neuron. Subconnectome neurons were removed from the inputs to the subconnectome neurons. For every neuron *n*, we compute the vector *n*_*in*_ of length equal to the number of neurons in the Hemibrain, with the *i*th entry equal to the synapse weight from neuron *i* to neuron *n*. If neuron *i* is not synaptically connected to neuron *n*, the corresponding entry is 0. For two vectors *u*, *v* the cosine distance is given by *d*_cos_(*u*, *v*) = 〈*u*, *v*〉/||*u*|| ||*v*||. Because all entries in *n*_*in*_ are nonnegative, cosine similarity ranges between 0 and 1. A similarity of 0 means that two neurons receive inputs from completely disjoint sets of neurons; a similarity of 1 means they receive inputs from exactly the same neurons in exactly the same proportion.

Mean similarity was computed within modules by averaging over all pairs of distinct neurons belonging to that module, and across modules by averaging over all pairs with one neuron in each cluster. For comparison, we ran 100 trials with shuffled module membership, preserving the size of each module but randomly assigning module labels to each neuron. The *z*-scores were computed from the distributions of mean pooled similarity of the shuffled data relative to the mean pooled similarity of the true data.

## Results

### oviIN is an outlier in the oviposition circuit

We queried the Hemibrain (Scheffer et al., 2020) for the oviposition cell types from Wang et al. (2020; Fig. 1*A*) and found the quantity of presynaptic and postsynaptic connections made by each of the oviposition cell types (Fig. 1*B*). The left instances are sometimes truncated and less complete than the right instances, thus left instances of oviposition cell types were excluded from the results. We were immediately struck by the oviIN because it makes far more synaptic connections than the other oviposition cell types (Fig. 1*B*). Particularly relevant is the quantity of input connections to oviIN. Even when excluding connection strengths <3, the right-hemisphere oviIN (oviIN_R) has 19,882 synaptic input connections which is far more than the quantity of inputs received by any other oviposition neuron.

The oviIN makes abundant connections within the oviposition circuit (Fig. 1*C*), particularly with SMP550 which is presumed to be the oviEN (Nojima et al., 2021). The oviIN and oviEN are strongly, reciprocally connected. As expected, oviIN is presynaptic to all subtypes of oviDN (including the putative additional pair of oviDNa that are labeled as SLP410 in the Hemibrain; Nojima et al., 2021) where it inhibits the initiation of the oviposition sequence. Among the pC1 neurons, oviIN receives inputs from all subtypes a–e and also sends outputs to subtypes b–e, forming a small recurrent motif. In accordance with whole-cell recordings from Wang et al. (2020), the SAG neurons strongly contact the pC1a-b neurons. SAG neurons also make moderately strong connections to pC1c in the Hemibrain data. The pC1a-b neurons make moderate connections onto the oviIN. Interestingly, the oviIN provides some feedback connections to the pC1b neurons (the 1 synaptic connection to one of the pC1a neurons was below the synaptic strength threshold of 3 for Fig. 1*C*). The remaining pC1 neurons are not among the subtypes that are believed to directly convey mating status to the oviposition neurons, but as noted elsewhere, the pC1 neurons are recurrently connected and may share mating status information received by the pC1a-b neurons (Deutsch et al., 2020; Wang et al., 2020). We note that pC1c sends relatively weak connections to oviIN but receives relatively strong feedback from oviIN. The connections that pC1d-e neurons make onto oviIN are stronger than those from pC1a-b onto oviIN. Although the pC1d-e neurons receive input from SAG neurons, they were not found to be responsive to SAG activation by Wang et al. (2020). The pC1d-e neurons also receive feedback from oviIN.

While oviIN forms strong synaptic connections with many of the oviposition cell types (we define strong synaptic connections among cell types as ≥ 100 connection weights), overall connections with the oviposition neurons make up a very small proportion of oviIN’s synapses (Fig. 1*D*). Roughly 3% of synapses on oviIN_R are from other oviposition neurons. It is clear from the Hemibrain data that oviIN makes a vast number of connections outside of the oviposition circuit. Furthermore, there is little overlap among the inputs that oviIN_R receives and those received by the right instances of oviEN (SMP550) and the oviDN subtypes (Extended Data Fig. 1-1). If we only consider connections with a weight of 3 or greater, we find that oviIN_R and SMP550_R have 37 presynaptic partners in common, which is a small portion of the presynaptic partners to SMP550_R (235) and to oviIN_R (1180). Only 3 of those 37 overlapping partners are cell types making strong inputs to oviIN_R. Since oviIN makes so many of its connections outside of the oviposition circuit, and it receives inputs that are largely distinct from those received by oviEN and oviDN, we chose to focus on analyzing the connectivity of oviIN—particularly its inputs.

### oviIN is an outlier in the brain

Given that oviIN’s extensive connectivity makes it an outlier in the oviposition circuit, we next sought to determine whether it is an outlier among the rest of the neurons in the Hemibrain with an eye toward other large inhibitory neurons. To eliminate possibly erroneous connections, we excluded connection weights of <3 between pairs of neurons from consideration in the subsequent rankings. We found that the oviIN is one of the most synaptically endowed neurons in the Hemibrain. The right oviIN is the seventh highest ranking neuron for total synaptic connections (47,044 synapse weights; Figs. 1*B* and 2*A*). The anterior paired lateral (APL) neuron has the most synaptic connections (228,713 synapse weights) out of all the 21,739 traced, uncropped neurons in the Hemibrain dataset. The APL has a role in normalizing the activity of odor-encoding Kenyon cells in the MB (Pitman et al., 2011; Prisco et al., 2021). In the synaptic connection ranking, it is closely followed by the right dorsal paired medial (DPM) neuron which is involved in odor memory and associative learning (Waddell et al., 2000; Keene et al., 2004, 2006), and is sleep-promoting (Haynes et al., 2015). The local lateral neurons (lLNs) of the antennal lobes also rank highly for synaptic connections. Notably, these highly synaptically endowed neurons are large inhibitory neurons (Chou et al., 2010; Pitman et al., 2011; Haynes et al., 2015). The median neuron in the Hemibrain makes a total of 597 synaptic connections. When considering the extent of oviIN_R’s inputs separately, it ranks 14th for synaptic input connections (19,882 synapse weights), making it an outlier in terms of the amount of input it receives.

The right oviIN ranks fifth for the number of total synaptic partners (pre and post) with 2,460 distinct partnering neurons (Fig. 2*B*), which is well above the median number of partners for neurons in the Hemibrain (76). APL ranks second with 2,775 partners, while the DPMs and lLNs have 2,411 and 2,264 on average, respectively. When considering only presynaptic partners, oviIN ranks 23rd while APL ranks 1st. However, the oviIN_R receives inputs from 1,180 neurons in the Hemibrain while the median neuron in the Hemibrain only receives inputs from 43 neurons.

The oviIN distinguishes itself from these other large inhibitory neurons with the number of cell types that it interacts with. With 943 cell types among its synaptic partners, oviIN ranks second in the Hemibrain on that metric (Fig. 2*C*). Despite a high ranking for the total number of synaptic connections, APL only ranks 235th in terms of the number of cell types among its direct connections with 250 cell types. The DPMs and lLNs connect with 90 and 219 cell types on average, respectively, while the median neuron in the Hemibrain makes direct connections with 33 cell types (Fig. 2*D*). Notably, the oviIN_R ranks first when only taking into account the number of cell types that are presynaptic to it. The oviIN_R receives inputs from 536 cell types while the median neuron in the Hemibrain receives inputs from 16 cell types. Compared to the median neuron in the Hemibrain which makes connections in 2 of the 12 neuropil supercategories, oviIN_R connects twice as many. The right oviIN makes synaptic connections in five supercategories (Extended Data Fig. 5-1) with most of those connections occurring in the SMP.

### Characterizing the relationship between oviIN and its inputs

**Figure 3.**
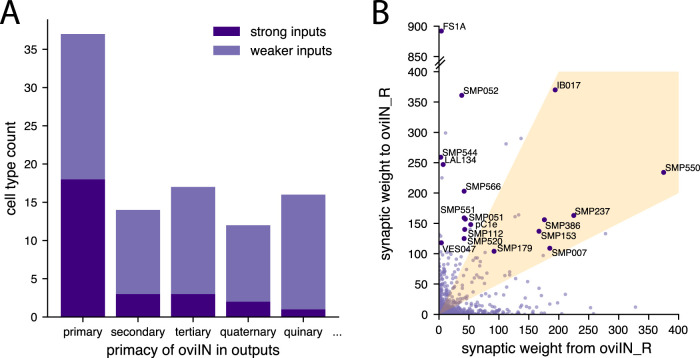
***A***, Cell types that have oviIN as their primary output also tend to be strong inputs to oviIN. Strong inputs make ≥100 synaptic connections to oviIN_R (dark purple); weaker inputs make <100 synaptic connections to oviIN_R (light purple). There are 37 cell types that have oviIN as their primary output. Among those, 18 cell types make 100 or more synaptic connections to oviIN_R. Cell types making strong connections to oviIN_R make up a smaller proportion of cell types that most strongly output to another cell type besides oviIN. ***B***, Each point on the graph represents a cell type that is a direct synaptic partner of oviIN_R. The combined synaptic weight from oviIN_R to a cell type is plotted along the *x*-axis while the combined synaptic weight to oviIN_R from a cell type is plotted along the *y*-axis. The 18 primary strong inputs to oviIN_R (dark purple points) are annotated with their cell type label. A subset of the primary strong inputs to oviIN_R occupy the zone of high reciprocity with oviIN_R (orange cone) where connections in the stronger direction are no more than twice the strength of connections in the weaker direction.

One could imagine that oviIN’s extensive connectivity might be a result of it receiving small amounts of input from many different neurons. On the contrary, we find that oviIN’s strongest presynaptic partners also favor oviIN as a postsynaptic partner (Fig. 3*A*). The oviIN_R is the primary target for the majority of cell types that strongly synapse onto it (with ≥100 synapse weights; 56.25 %). As might be expected, this primacy decreases for cell types that do not send strong connections to oviIN, indicating that those cell types prioritize making connections with other neurons besides oviIN. As a point of comparison, a much smaller portion of the strong inputs to the APL primarily send their synapses to APL (4.3%). Only the LHMB1 and LHCENT5 cell types are primary strong inputs to APL. This finding confers with APL’s divisive normalization function where it samples from many neurons to obtain a population-level average of activity. As such, the oviIN’s role does not appear to be divisive normalization. Rather, the primacy of the strong inputs to oviIN is suggestive of dedicated pathways of information flow.

**Figure 4.**
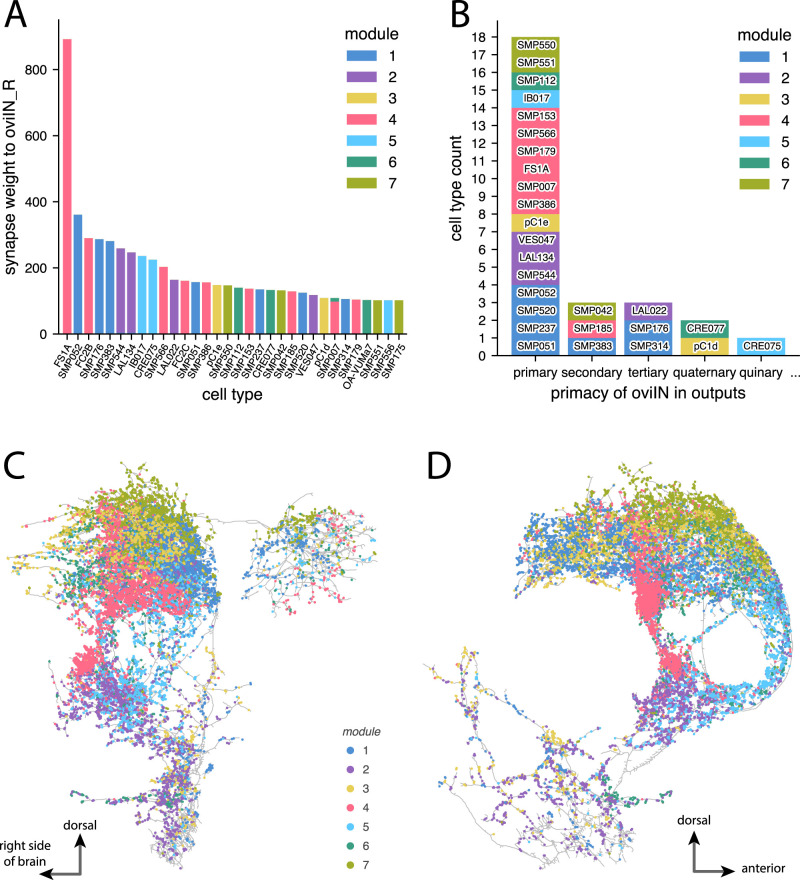
Modularity maximization reveals mesoscale and microscale connectome structure among inputs to oviIN_R. ***A***, The strongest inputs to oviIN_R. Neuronal cell types that make an aggregated synapse count of at least 100 onto oviIN_R are shown along the *x*-axis. The bars indicating synapse counts are color-coded by the module(s) into which the neurons of a type are clustered. Sometimes, neurons of the same type are clustered into different modules. For example, three instances of SMP007 are grouped into module 4 and the other instance is grouped into module 6 (there are four instances of SMP007, all on the right side of the brain). ***B***, Most of the strongest inputs to oviIN_R are primary inputs, meaning that the cell type primarily targets oviIN. Primacy indicates oviIN_R’s ranking among the cell type’s strongest outputs. Only cell types with aggregated connection strength ≥100 onto the oviIN_R are shown. Each cell type is colored according to the module where most of its instances are clustered. ***C***, Front view of synaptic input sites on oviIN_R colored by module. The reconstructed skeleton of the right oviIN from the Hemibrain is outlined in gray. Each dot on the body of oviIN_R is a postsynaptic site. These connection sites are colored according to the module of the presynaptic neuron. Synapses from neurons without a cell type label (annotated as “None”) are not shown, but results are qualitatively the same when they are included. See Extended Data Figures 4-1 to 4-7 for individual modules, Extended Data Figure 4-8 for neuropil supercategories where synapses are located, and Extended Data Figure 4-9 for module membership. ***D***, Lateral view (from the right side of the brain) of synaptic input sites on oviIN_R colored by module as in (**C**).

10.1523/ENEURO.0123-25.2025.f4-1 Figure 4-1 Synaptic input sites on oviIN_R from module 1. Left panel: front view; Right panel: lateral view. The 20 cell types from module 1 making the strongest connections to oviIN_R are shown in the legend. Download Figure 4-1, TIF file.

10.1523/ENEURO.0123-25.2025.f4-2 Figure 4-2 Synaptic input sites on oviIN_R from module 2. Left panel: front view; Right panel: lateral view. The 20 cell types from module 2 making the strongest connections to oviIN_R are shown in the legend. Download Figure 4-2, TIF file.

10.1523/ENEURO.0123-25.2025.f4-3 Figure 4-3 Synaptic input sites on oviIN_R from module 3. Left panel: front view; Right panel: lateral view. The 20 cell types from module 3 making the strongest connections to oviIN_R are shown in the legend. Download Figure 4-3, TIF file.

10.1523/ENEURO.0123-25.2025.f4-4 Figure 4-4 Synaptic input sites on oviIN_R from module 4. Left panel: front view; Right panel: lateral view. The 20 cell types from module 4 making the strongest connections to oviIN_R are shown in the legend. Download Figure 4-4, TIF file.

**Figure 5.**
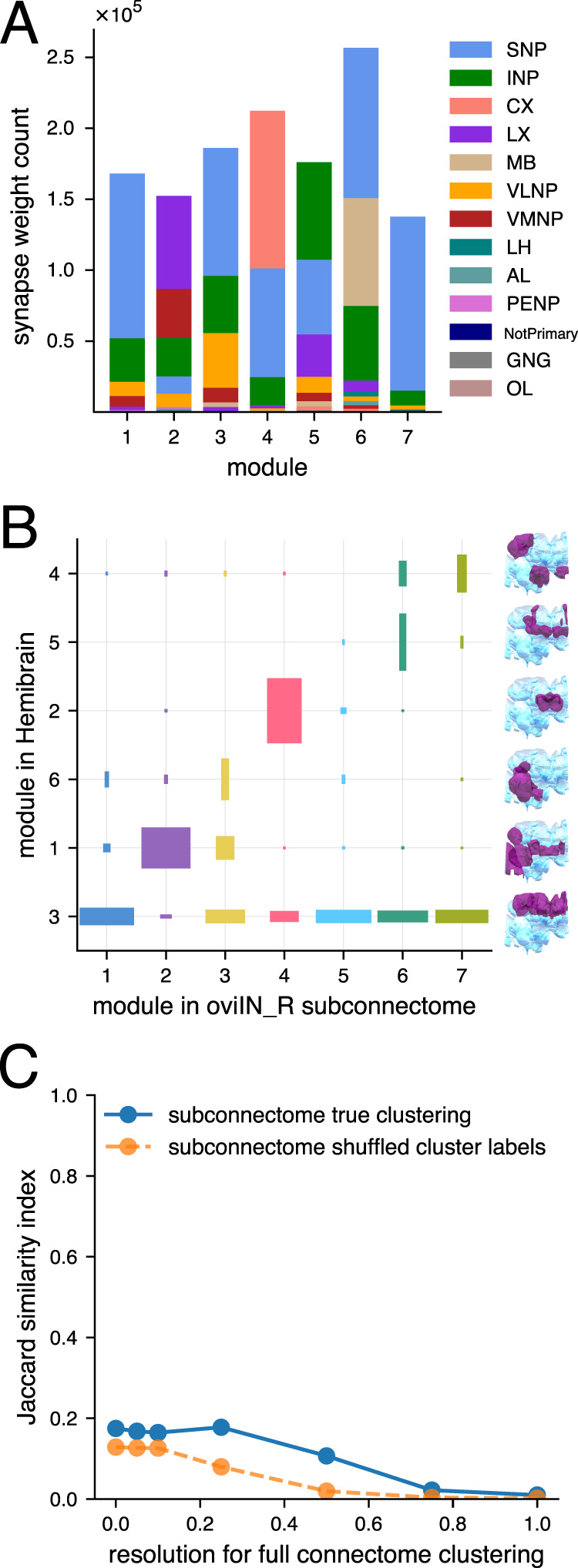
***A***, The counts of connections received within the various neuropil supercategories by the neurons collectively in each module. The neurons in most of the modules receive a large portion of their synaptic input in the SNP. There are some synapses that fall outside of the 63 primary neuropils that are defined in the Hemibrain data. As a result, those synapses cannot be labeled with a supercategory and are instead labeled as “NotPrimary.” We use the supercategories defined by Ito et al. (2014; Extended Data Fig. 5-1). ***B***, Modularity maximization returns partitions of the oviIN_R input subconnectome that are distinct from partitions for the full Hemibrain connectome. The seven modules that we found for the subconnectome of oviIN_R’s inputs are shown along the *x*-axis. The modules for the full Hemibrain found by Kunin et al. (2023) are on the *y*-axis, and an anatomical rendering is provided for each of those modules on the righthand side of the plot showing their most closely corresponding neuropils (purple) within the brain (light blue). The width and height of each box is an indication of the portion of nodes that jointly appear in a given module of the subconnectome clustering and a given module of the full connectome clustering. The colors correspond to the subconnectome clustering from Figure 4. ***C***, Jaccard similarity between the coarse clustering of the subconnectome of oviIN_R’s inputs and the full connectome at various clustering resolutions (solid blue line), from coarse (0.0 resolution) to fine (1.0 resolution). Jaccard similarity for a random baseline is plotted in orange (dashed line) where the cluster labels for the subconnectome were randomly shuffled before being compared to the full connectome clustering.

10.1523/ENEURO.0123-25.2025.f4-5 Figure 4-5 Synaptic input sites on oviIN_R from module 5. Left panel: front view; Right panel: lateral view. The 20 cell types from module 5 making the strongest connections to oviIN_R are shown in the legend. Download Figure 4-5, TIF file.

**Figure 6.**
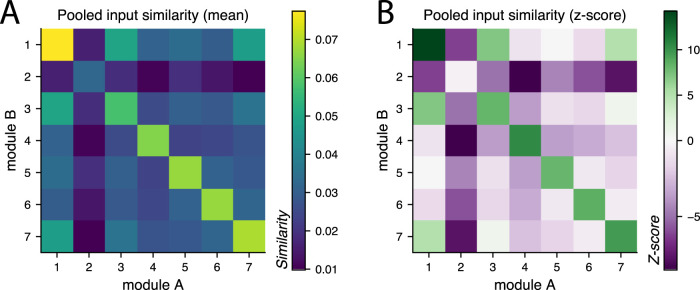
***A***, Mean pooled input similarity of the modules of the subconnectome. Subconnectome neurons have higher input similarity with neurons of the same module than with neurons from other modules (see diagonal). The inputs to neurons in the subconnectome of oviIN_R’s inputs include direct connections from neurons anywhere in the brain (except from neurons in the subconnectome). Heatmap colors indicate the mean input similarity between all neurons in module A versus module B. ***B***, *Z*-scores of pooled input similarity. Module labels were randomly shuffled (*n* = 100 shuffles) in a random model of input similarity. Along the diagonal, the true mean pooled input similarity was often several standard deviations away from that of the random model, indicating that the input self-similarity of the modules in the true clustering is not random. See full histograms in Extended Data Fig. 6-1.

10.1523/ENEURO.0123-25.2025.f4-6 Figure 4-6 Synaptic input sites on oviIN_R from module 6. Left panel: front view; Right panel: lateral view. The 20 cell types from module 6 making the strongest connections to oviIN_R are shown in the legend. Download Figure 4-6, TIF file.

10.1523/ENEURO.0123-25.2025.f4-7 Figure 4-7 Synaptic input sites on oviIN_R from module 7. Left panel: front view; Right panel: lateral view. The 20 cell types from module 7 making the strongest connections to oviIN_R are shown in the legend. Download Figure 4-7, TIF file.

10.1523/ENEURO.0123-25.2025.f4-8 Figure 4-8 Supercategories where synapses from presynaptic partners to oviIN_R are localized. For the neurons within each module, the counts of synapses to oviIN_R within a neuropil are used to compute the supercategory synapse weight counts (see *Figure 5-1*). Download Figure 4-8, TIF file.

10.1523/ENEURO.0123-25.2025.f4-9 Figure 4-9 Cell types in each module of the subconnectome of oviIN_R’s inputs. The module is indicated in the second column. The third column contains the total synapse weight onto oviIN_R by the neurons of the indicated cell type in the indicated module. Download Figure 4-9, CSV file.

10.1523/ENEURO.0123-25.2025.f5-1 Figure 5-1 All Hemibrain neuropils from the v1.2.1 dataset along with their supercategories as described by ***Ito et al.*** (***2014***). The colors of each row correspond to the supercategory colors used in *Figure 5*A and *Figure 4-8*. A check mark in the last column denotes a neuropil where the oviIN_R receives connections. Download Figure 5-1, TIF file.

The primary strong inputs to oviIN lay the foundations for potential pathways to oviIN from other circuits, and their identities may be crucial for determining which circuits they funnel information from. However, the majority of cell types connecting to oviIN are uncharacterized, meaning they have either not been studied before being identified in the Hemibrain or they have not yet been identified from existing scientific literature. The Hemibrain provides generic labels to uncharacterized cell types that indicate the neuropil in which the bulk of their synapses are found (Scheffer et al., 2020). Neurons that have similar morphology and significant overlap in their synaptic connection patterns are assigned to the same cell type in the Hemibrain. A large proportion of the inputs to oviIN, including its primary strong inputs, are cell types with Hemibrain-provided generic cell type labels that indicate that they mainly reside in the SMP. However, oviIN’s strongest input comes from the FS1A cell type. The FS1A neurons are a columnar output cell type of the fan-shaped body (FB; Hulse et al., 2021). The FS1A neurons mainly receive FB inputs from layers 2 to 6 and leave the FB to project outputs to the SMP and crepine. The pC1d and pC1e are also among the strongest inputs to oviIN. The pC1d has been shown to promote aggressive behavior in the female fly (Schretter et al., 2020), while activation of pC1d-e together drives a persistent response of several minutes indicative of an internal state encoding (Deutsch et al., 2020).

Among the primary strong inputs to oviIN, some of the cell types form highly reciprocal connections with oviIN_R while others have more of a feedforward relationship with it (Fig. 3*B*). The FS1A, SMP544, LAL134, and VES047 cell types hardly make any feedback connections to oviIN_R. In contrast, the SMP179, SMP386, SMP153, SMP237, SMP550, SMP007, and IB017 cell types are strongly reciprocal with oviIN_R. These findings imply that FS1A, SMP544, LAL134, and VES047 neurons directly convey information from other circuits while the information conveyed by oviIN’s reciprocally connected primary strong inputs, which are primarily uncharacterized SMP types, can be modulated by oviIN.

### Clustering the inputs to oviIN

The FS1A and pC1 neurons are not known to have any overlapping functions, suggesting that oviIN integrates information from at least two distinct circuits. However, the right oviIN receives inputs from 943 cell types leading us to think that many of those cell types might be associated with the same circuits. Furthermore, there is the problem of what to make of the hundreds of uncharacterized cell types among the inputs to oviIN. To begin to organize the inputs projecting to oviIN, we used a network community-detection method. We employed a machine learning algorithm, RenEEL (Guo et al., 2019; see Methods and Materials), to find clusters of densely connected neurons from an undirected network where the nodes are neurons and the links are weighted synaptic connections.

RenEEL maximizes modularity which is a measure of the excess of within-group connections compared to a randomly connected network (Newman, 2006). The goal of maximizing modularity is to find an optimal partitioning of the network into modules (i.e., clusters) with higher within-group than between-group connectivity. Generalized modularity analysis is a multi-resolution method that can find community structure at various scales by adjusting a resolution scale parameter (Guo et al., 2023). At a low resolution, the coarse structure of the network is returned as a small number of modules with many neurons in each. As the resolution is increased, the network is partitioned into a greater number of smaller modules. The clusters of densely connected neurons identified in this way may form the basis of a hierarchical circuit structure. Previous studies identified community structure in the full Hemibrain connectome at multiple resolutions by maximizing generalized modularity density (Eq. 1; Kunin et al., 2023). This unsupervised analysis recovered the gross structure of the brain by returning modules that reflect the neuropil super-structures. At higher resolutions, known fine structures, such as the sub-circuits of the layered FB, were recovered.

In the current study, we limit our analysis to classic modularity (coarse resolution) of the network of inputs to the right oviIN. Since this network is a part of the full connectome, we refer to it as a subconnectome. The subconnectome of inputs to oviIN_R contains all of oviIN_R’s presynaptic partners and the connections among them, and it excludes all other connectome neurons. Our goal was to identify community structure that may otherwise be subsumed by the super-structure of the neuropils when analyzing the full Hemibrain connectome. The modular structure we uncover depends on the relative connection strengths within and between groups; thus, by eliminating neurons that do not form direct connections to oviIN_R we can hope to identify densely connected communities that potentially span multiple brain areas. RenEEL returned seven modules containing from 199 to 347 neurons each for the subconnectome of inputs to oviIN_R (Extended Data Fig. 4-9). Each module contains between 111 and 199 unique cell types.

### The modules divide the primary strong inputs to oviIN_R

The most prominent inputs to oviIN_R are spread out among the different modules (Fig. 4*A*), indicating that they represent parallel pathways that funnel distinct information to oviIN_R. The FS1A cell type is clustered into the same module as the other FB cell types that send strong inputs to oviIN (FC2B and FC2C; module 4) along with select, uncharacterized SMP cell types. The pC1d-e are clustered together in module 3. The primary strong inputs to oviIN_R are also spread among the modules (see primary bar in Fig. 4*B*), supporting the notion that if oviIN is a hub, the primary strong inputs define the spokes that convey information from distinct circuits.

Although most of the cell types in these modules are uncharacterized, there are a few characterized cell types that corroborate the potential functional basis of the modules. The aIPg1-3 cell types were previously found to elicit aggressive behavior from female flies and to form a recurrent network with the pC1d-e (Deutsch et al., 2020; Schretter et al., 2020). The aIPg cell types make moderate connections to oviIN, and they are clustered together with the pC1d-e in module 3 (Extended Data Fig. 4-9). We note that pC1a-b are clustered into module 7 with the oviEN (SMP550) while the pC1c are in module 1. Module 7 has other *fru+* (fruitless-expressing) neurons that, along with the pC1a-b neurons, are broadly implicated in female receptivity. These include vpoDN (Wang et al., 2021), SAG (Feng et al., 2014), and a non-fruitless cell type, SMP286 (a.k.a. pCd-2; Imoto et al., 2024). Also in module 7 is the SMP029 which is a candidate for the aDN (Matsliah et al., 2023) which have a role in site selection during oviposition (Nojima et al., 2021).

These examples are only a handful of neurons and cell types from among the hundreds of neurons in their respective modules and therefore it would not be reasonable to ascribe a specific function to the entire module based on the minority of cell types that happen to be characterized. However, it is reassuring that characterized neurons with related functions were found in the same module. Furthermore, the relationships between characterized neurons and the primary strong inputs to oviIN in their module may provide clues about the functions of those uncharacterized primary strong inputs.

The only clock neurons that directly connect to oviIN are two LPN and two instances of the LNd cell type which correspond to the E1 subtype of evening cells (Shafer et al., 2022). These neurons are clustered together in module 1 with aMe24, a cell type from the accessory medulla, an area involved in circadian rhythms (Helfrich-Förster et al., 2007). Module 1 is also where the left oviIN was found. The E1 LNds have reciprocal connections with the SMP520 which is a primary strong input to oviIN that was clustered into module 1 (Fig. 4*B*). Whether or not the SMP520 carries information about circadian timing to oviIN remains to be seen because the LNd inputs (indeed, any clock inputs) to SMP520 are not among its strongest inputs. However, three other primary strong inputs from module 1, SMP237, SMP051, and SMP052, make a small, strongly recurrent network with the aMe24 and pC1c.

Modules 5 and 6 are peppered with MB output neurons (MBONs). The three MBON cell types in module 5 (MBON27, MBON33, and MBON35) are atypical MBONs, meaning they receive inputs from outside the MB (Li et al., 2020). MBON27 is specialized for visual inputs (Li et al., 2020), and it is implicated in a small network connecting CX to MB along with oviIN and mALD1 which appeared in module 5 as well (Hulse et al., 2021). The sole primary strong input to oviIN_R from module 5, IB017, makes relatively weak reciprocal connections with mALD1 and barely connects to the MBON27, indicating that it is not part of that CX to MB circuit. However, IB017 does form moderate connections with MBON35 and MBON33. The MBONs in module 6 are a mixture of typical and atypical types (MBON01, MBON04, MBON05, and MBON12; atypical: MBON26, MBON31, and MBON32). The connectivity profile of the MBON12 suggests that it has a role in water-seeking behavior, and MBON26 receives a significant amount of thermo-hygrosensory input (Li et al., 2020). The SMP112, the only primary strong input to oviIN_R in module 6, does not receive strong inputs from MBON12 and MBON26 but it does receive moderate input from MBON31 and MBON32 which are the strongest inputs to MBON26.

As mentioned, the FB cell types that project to oviIN_R were mainly clustered into module 4. The FS1A, FC2B, and FC2C form a strongly recurrent network. The SMP cell types in module 4 that are primary strong inputs to oviIN_R make very sparse connections to these FB cell types, but the SMP386, SMP153, and SMP007 receive moderate to strong inputs from the FB cell types, indicating that the flow of information goes from the FB neurons to the recurrently connected network of oviIN, SMP386, SMP153, and SMP007. In particular, oviIN and FS1A are the strongest cell type inputs to SMP386. FS1A is the strongest primary input to oviIN, but the SMP386, SMP153, and SMP007 are also primary strong inputs to oviIN and they may form a secondary pathway through which FB information can reach the oviIN, or they may form a circuit that performs a distinct computation on FB inputs before conveying the results to the oviIN.

### Anatomical organization of the modules

RenEEL was run 30 times on the same subconnectome data with different random seeds and it largely returned the same modules. While this gives us confidence in the RenEEL algorithm’s ability to detect fairly consistent communities within the network of oviIN_R’s inputs, it does not on its own confirm that RenEEL has uncovered biologically relevant brain structure. To determine whether there is any spatial organization inherent in the modules, we visualized the input synapses to oviIN_R on its reconstructed skeleton (Fig. 4*C, D*; Extended Data Figs. 4-1–4-7). The input synapses on oviIN_R are shown as dots and each dot is colored according to the module of the presynaptic neuron. Neurons from modules 1, 3 and 7 mostly synapse onto oviIN_R in the dorsal tuft of processes within the SMP where most of oviIN_R’s output sites also reside (Extended Data Figs. 4-1, 4-3, 4-7). Neurons from modules 4 and 6 also form most of their synapses onto oviIN_R in the dorsal tuft, but they also have a large portion of synapses in the inferior neuropils (INP) along the posterior and anterior part of the crepine, respectively (Extended Data Figs. 4-4, 4-6). Module 5 neurons predominantly form their synapses to oviIN_R along the anterior crepine, but also occupy portions of the dorsal tuft (Extended Data Fig. 4-2). Neurons from module 2 mainly form their connections along the ventral portion of the oviIN_R intersecting with lateral complex (LX), ventromedial (VMNP) and INPs (Extended Data Fig. 4-8). The modules do not simply reflect the boundaries of the neuropils that intersect with oviIN_R because all of the module synapses intersect with a mixture of neuropils and they all intersect to some extent with the SNP (Extended Data Fig. 4-8). Nonetheless, the anatomical organization of modules indicates that RenEEL found biophysically meaningful clusters of neurons. The organization along the distinct branches of oviIN_R suggests that neurons work together with the other neurons in their module to influence the activity within different regions of this large neuron. It is possible that the motifs within these modules act as local processing units to drive, summate, or shunt activity from other local processing units.

It is reasonable to wonder whether the anatomical organization of the modules is a reflection of the neuropil organization of the brain more broadly, rather than the neuropil boundaries of the synapses on oviIN_R. In other words, has RenEEL clustered oviIN_R’s inputs according to the neuropils that they “belong to”? Strictly speaking, neurons cannot easily be classified as belonging exclusively to one neuropil because they often make connections in multiple neuropils, although researchers sometimes classify a neuron based on the neuropil where it makes the majority of its synaptic contacts (Ito et al., 2014; Scheffer et al., 2020; Schlegel et al., 2024). To determine the various brain regions that are represented among the inputs to oviIN_R, we queried the Hemibrain for the neuropils in which oviIN_R’s presynaptic partners receive synaptic connections from any neuron. We found that RenEEL did not simply partition oviIN_R’s input connectome according to the neuropils where those neurons receive their connections (Fig. 5*A*). In all but modules 2 and 5, the neurons receive the majority of their synapses in the SNP. We had already determined that, collectively, the majority of input connections to oviIN_R are made in the SNP (Extended Data Fig. 4-8), and that a large proportion of oviIN_R’s input connectome contains neurons that are labeled as SMP neurons. Figure 5*A* shows that RenEEL has identified SNP sub-groups that may pertain to distinct circuits in the SNP. Neuropil designations are primarily driven by anatomical considerations and do not necessarily demarcate functional circuits (Yu et al., 2013; Ito et al., 2014). Thus, the modules may loosely represent circuit structure that draws from SNP together with other neuropils. For example, the most prominent cell type input to oviIN, FS1A, is in module 4. The FS1A neurons receive most of their input connections in the FB neuropil of the CX, but they make most of their output connections in the SMP (of the SNP) and crepine (of the INP; Hulse et al., 2021). Cell types such as this may form a bridge from one part of the brain to the SNP, INP, LX, or VMNP neuropil supercategories where they interact with oviIN.

### Functional organization of the modules

We next wondered whether the partitions found for oviIN_R’s input connectome reflect those previously found for the full Hemibrain connectome by Kunin et al. (2023). The coarse modules they found in the full Hemibrain connectome were roughly aligned with the neuropil structure of the brain, indicating that RenEEL identified macroscale brain structure. Even though we only consider neurons which make direct synaptic contact with the right oviIN in the present study, we might expect strong alignment with the full Hemibrain modules if the brain’s super-structure is still present within the subconnectome of oviIN_R’s inputs. We compared our results to the modules identified by Kunin et al. (2023), and we did not find strong alignment. The portion of oviIN_R’s input connectome that resides in full Hemibrain module 3—which corresponds mostly to SMP and SIP neuropils—is spread out among all of the subconnectome modules (Fig. 5*B*). In other words, large portions of the neurons in the subconnectome modules all share a common module in the full Hemibrain clustering—namely the module that encompasses the medial and intermediate superior neuropils. Full Hemibrain module 1 corresponds mainly to the VMNP, and the portion of oviIN_R’s connectome that is from that area is dominated by subconnectome module 2. Likewise, full Hemibrain module 2 (which corresponds to FB) is mainly represented in subconnectome module 4. However, all of the subconnectome modules are composed of a combination of the full Hemibrain modules. Since every cluster identified in oviIN_R’s connectome has significant overlap with the full Hemibrain module that encompasses the SIP and SMP, this suggests that, in the full Hemibrain, the connections within SIP and SMP are stronger overall than connections between these areas and other neuropils, and that connections within potential circuits that include SIP and SMP neurons combined with other cell types are dense enough to be detected in the absence of the connections with the broader network of each neuropil.

To determine whether the coarse modules of oviIN_R’s input connectome correspond to mid-resolution modules of the full Hemibrain, we computed the Jaccard similarity (Eq. 3) between the coarse clustering of the subconnectome of oviIN_R’s inputs and the clusterings of those same neurons in the full connectome at various resolutions. A high Jaccard similarity (close to 1) is an indication that many pairs of neurons are partitioned into a module together in both the subconnectome clustering and the full connectome clustering at a particular resolution. We found low similarity between the subconnectome clustering and the full connectome clustering (Fig. 5*C*). For all resolutions of the full connectome clustering, Jaccard similarity with the subconnectome clustering was <0.2. However, Jaccard similarity was always slightly higher than a random baseline in which the module labels for the subconnectome were shuffled. Thus, applying RenEEL to a subconnectome of the brain, as we have done for oviIN_R’s input connectome, does not partition the neurons in the same way as for the full connectome, either at the coarse or higher resolutions. Our results suggest that the modules correspond to a microscale organization at the level of oviIN_R’s inputs—which may correspond to distinct circuit pathways and which may have functional significance for oviIN.

To verify that these modules are representative of distinct pathways that converge onto oviIN_R, we once again considered their connections within the full Hemibrain. We computed the similarity of inputs from anywhere in the brain to each pair of neurons in oviIN_R’s input connectome (see Methods and Materials). On average, the similarity of within-module pairs exceeds those of between-module pairs (Fig. 6*A*). In other words, presynaptic partners of oviIN_R have more of their inputs in common with other neurons in their same module than with neurons in a different module. Moreover, comparison to a randomly partitioned network reveals that within-module pairs are more similar than would be seen by chance (Fig. 6*B* and Extended Data Fig. 6-1). Taken together, these results support the idea that the modules approximate parallel streams of input from different circuits.

10.1523/ENEURO.0123-25.2025.f6-1 Figure 6-1 Randomization of module labels (n = 100 shuffles) shows decreased similarity for self-same cluster pairs. Input similarity is shown on the *x*-axes (similarity ranges from 0 to 1). The counts of similarity values are shown on the *y*-axes. The red dashed line in each plot indicates the mean input similarity between modules as visualized in *Figure 6*A. The histograms show the similarity scores for the random models. Along the diagonal, the mean input similarity among neurons in the same module (red dashed line) tends to be higher than the mean input similarity among neurons from random modules. In off-diagonal plots, the mean input similarity between neurons from two different modules (red dashed line) tends to be within the similarity distribution for neurons from random modules or lower than the distribution mean. Download Figure 6-1, TIF file.

## Discussion

With a central nervous system consisting of just under 140,000 neurons (Dorkenwald et al., 2024), the fruit fly, *Drosophila melanogaster*, exhibits the capacity for complex behavior driven by neuronal circuits that carry out sophisticated brain computations (Griffith, 2013; Haberkern and Jayaraman, 2016; Scheffer and Meinertzhagen, 2021; Oram and Card, 2022). Context-dependent computations, which conditionally process or integrate inputs, endow an animal with behavioral flexibility (Devineni et al., 2019; Hindmarsh Sten et al., 2021; Cury and Axel, 2023; Roemschied et al., 2023). For instance, hungry flies persistently track a food odor despite repeated failure to find food, showing that need is selectively reinforced in such conditions (Sayin et al., 2019). Context can modulate egg-laying preference for acetic acid, despite the otherwise strong avoidance for this as food (Joseph et al., 2009). Additionally, circadian rhythms influence egg-laying, making oviposition more likely around the onset of night (Allemand, 1976; Cury et al., 2019; Riva et al., 2024). Processed sensory inputs integrate with context, memory, and state, in what we term high-level processing, to produce complex behavior.

The complexity inherent in the decision-making and behavior of *Drosophila* makes studying its tiny brain a worthwhile endeavor because isolating the circuits that drive high-level processing is within reach. The *Drosophila* brain has a hierarchical organization where peripheral sensory inputs undergo multiple stages of higher-order processing, evident in both the adult (Jeanne et al., 2018; Kunin et al., 2023) and larval brain (Winding et al., 2023; Betzel et al., 2024). Researchers have made progress studying the sensory periphery and progressively discovering higher-order neurons and circuits (Jacobs et al., 2024; Matsliah et al., 2024; Walker et al., 2024), but such an approach is inadequate for discovering the circuits that drive the complex computations involved in context-dependent decision-making. Behaviors like oviposition depend on nearly every sensory modality, and starting at the periphery to discover the high-level circuits that integrate sensory inputs, state, and memory would require an expansive search. The connectome now offers another way to discover the neural circuits that are responsible for high-level processing. Although we have not identified verifiable circuits, our study takes a first step toward that goal.

The Hemibrain data has primarily been used to study well-characterized regions of the brain such as the CX (Hulse et al., 2021) and the MB (Li et al., 2020). The cell types and connectivity profiles of the CX circuits that compute abstract internal representations of angular orientation, maintained by self-motion signals and visual cues, have been cataloged by Hulse et al. (2021). Furthermore, they found that the output types of the CX project to various parts of the brain, including premotor and sensory areas, and that they also return to the CX, evoking a deep recurrent network. The MB supports associative learning, enabling flies to pair odorants with positive and negative experiences (Aso et al., 2014a,b; Handler et al., 2019). Investigations using the connectome and in vivo calcium imaging have led to a detailed understanding of how the APL neuron normalizes Kenyon cell signals within the MB (Prisco et al., 2021). The connectome has also aided researchers in discovering new MB cell types that project outside of the MB lobes (Li et al., 2020) and that receive visual inputs as well as odor information (Li et al., 2020; Ganguly et al., 2024). While great advances have been made in understanding these well-studied areas of the brain, there remains significant potential to leverage this dataset to investigate less well understood regions (Ito et al., 2014; Scheffer and Meinertzhagen, 2021; Lin et al., 2024).

In our approach, we analyzed the inputs to the oviposition circuit to study the neural correlates for the highest level computations that drive oviposition. We first observed that the oviIN is an outlier in the oviposition circuit and in the brain due to its abundant connectivity within and beyond the oviposition circuit. Given that the vast majority of inputs to oviIN are uncharacterized cell types, our goal was to identify the structural pathways into the oviposition circuit so that future work could determine the sensory and functional bases for these input pathways. We found that oviIN_R is the primary target of multiple cell types rather than solely collecting small amounts of input from neurons that primarily send outputs elsewhere. This pattern of connectivity is more aligned with a multi-circuit integrator role than a divisive normalizer. After applying a community-detection analysis, we found that the primary strong inputs to oviIN_R are distributed across multiple modules indicating that those primary strong inputs form the backbones of multiple distinct pathways to oviIN. Furthermore, we show that our modularity results do not reflect the same scope of anatomical organization that was found in the full Hemibrain modules by Kunin et al. (2023). Instead, maximizing modularity for the subconnectome containing oviIN_R’s inputs reveals an anatomical organization at a biophysically relevant scale for oviIN_R that is reminiscent of dendritic processing. Finally, we show that there is higher similarity among the inputs to neurons within a module than across modules, which further supports our interpretation of the modules as partitions around pathways that convey distinct information to oviIN.

Our study is limited to a partial brain connectome of a single specimen. Variability among individual female fruit flies may hinder the generality of our results, as well as the missing connections with neurons on the far left side of the central brain in the Hemibrain data. Additionally, the Hemibrain connectome only reconstructs classical chemical synaptic connections. Although electrical synapses and neuropeptides play an important role in the computations and functions of the *Drosophila* brain, gap junctions and neuropeptide release sites are not included in the current connectome data. Future iterations of *Drosophila* connectomes may include these additional features, enabling us to build upon the results we present here.

### High-level processing in the SNP

The majority of oviIN’s inputs come from the SNP—an understudied region that includes the SLP, SIP, and SMP neuropils and that lacks a topological organization (Ito et al., 2014). These unstructured regions contain the components of circuits related to oviposition (Wang et al., 2020; Zhang et al., 2020; Vijayan et al., 2023), circadian rhythms (Kaneko and Hall, 2000; Fernández et al., 2008; Shafer et al., 2022; Reinhard et al., 2024), olfaction (Ruta et al., 2010; Séjourné et al., 2011; Aso et al., 2014a; Wang et al., 2014; Cohn et al., 2015), and social behaviors like courtship and aggression (Zhou et al., 2014; Deutsch et al., 2020; Schretter et al., 2020; Wang et al., 2021; Taisz et al., 2023; Coleman et al., 2024). The diffuse neuropils of the Protocerebrum are rife with high-level processing potential. MBONs carry odor information and send projections to the SNP at convergence zones where they meet the dendrites of dopaminergic neurons which respond to the valence of a stimulus (Aso et al., 2014a,b). Thus, the SNP are likely sites for circuits that are responsible for classical conditioning. Sensory and interoceptive inputs to the subesophageal zone are relayed to the SMP where they regulate water and food seeking behavior (Jourjine et al., 2016; Landayan et al., 2021; González Segarra et al., 2023). Taste projection neurons that project to the SLP are essential for conditioned taste aversion whereas those that bypass the SLP are not (Kim et al., 2017). The sexually dimorphic pC1 neurons have a role in persistent states related to courtship and aggression (Zhou et al., 2014; Deutsch et al., 2020). They are presynaptic to oviIN, and according to the Hemibrain, they make most of their synapses in the SNP as well. Despite these behaviorally relevant functions, the SNP remain *terra incognita* as far as its organization (Ito et al., 2014; Scheffer et al., 2020).

Without a pre-existing map of the SNP or a comprehensive wiring logic, we used modularity maximization to get a first approximation of the organization inherent in the abundant inputs to oviIN. To be clear, we do not claim to have identified complete (nor partial) circuits since we intentionally limited our modularity maximization to the network of neurons that are directly presynaptic to oviIN_R. Rather, our coarse clustering cast a wide net around the primary strong inputs to oviIN to dredge up clues about the regions and circuits that are associated with its inputs. Being able to associate uncharacterized cell types with each other, or with known cell types, is a far cry from discovering novel circuits, but it is a reasonable first step given that the SNP form abundant reciprocal connections among themselves and with other neuropils (Lin et al., 2024). This approach enabled us to determine to what extent the primary strong inputs are independent “spokes” of a hub. Instead of the primary strong inputs coalescing into a single module, we found that there are primary strong inputs in every module. This suggests that there are distinct streams of inputs converging onto the oviIN. Ultimately, our approach revealed some approximate organization among oviIN’s inputs that may support the discovery of the circuits that are involved in the integration of sensory information, and the computations of evaluation and decision-making in oviposition. Future work will be needed to uncover and characterize the potential circuits that our connectivity analysis alludes to.

We observed that the FS1A neurons form recurrent connections among themselves and with other FB types, and they project to a recurrent network of SMP neurons that includes SMP386, SMP153, and SMP007. The FS1A neurons also project directly and strongly to oviIN. Thus we found two pathways along which FB information may flow to oviIN: a direct pathway via FS1A, and an indirect pathway from FS1A through a recurrently connected network of uncharacterized SMP neurons which themselves make strong, direct connections to oviIN. This suggests that the recurrently connected SMP neurons that are targeted by FS1A perform a distinct computation on the FB information received. Furthermore, since these SMP neurons also form recurrent connections with oviIN, they may be involved in a computation of FB input that is itself modulated by oviIN. It is possible that the SNP are organized into recurrently connected microcircuits such as this that perform high-level computations.

### Selective application of modularity analysis for structural scope

Network community-detection methods generally fall into two classes: spectral methods which cluster nodes by leveraging the spectral properties of the graph to embed the network into a low-dimensional space (Chung et al., 2021), and modularity-based methods which partition nodes to maximize the difference between observed intra-cluster connections and those expected under a random graph model.

We used a modularity maximization approach using the RenEEL machine learning scheme (Guo et al., 2019) because of its ease of interpretation. Its emphasis on intra-connectedness of the node communities (Newman and Girvan, 2004; Newman, 2006; Guo et al., 2023; Kunin et al., 2023) reveals assortative groups of neurons and cell types. By forming densely connected modules within the subconnectome of oviIN_R’s inputs, our modularity-based method offers insight into the structural organization of this network and provides predictions for the functions of the cell types that are clustered together.

In our study, we chose to apply RenEEL to a subnetwork of the connectome to control the scope of the structure we were probing. RenEEL was previously applied to the full Hemibrain connectome by Kunin et al. (2023) and had recovered gross anatomical structure that reflects the neuropil organization of the brain, as well as finer structure such as the layers of the FB. While such macroscale anatomical structure often underlies functional circuit structure (Betzel et al., 2013), it might obscure mesoscale and microscale circuit structure that is not necessarily contained within a given neuropil (Betzel et al., 2018; Betzel, 2023). For example, the FS1A and the SMP386 neurons are from different neuropils and they are clustered into separate modules for the full Hemibrain connectome; however, they are clustered into the same module when RenEEL is applied to the subconnectome of oviIN_R’s inputs. These neurons are separately partitioned in the full connectome because the connections they make with other neurons from their own neuropil are denser than any connections made with neurons outside of their neuropils. When the scope of the network is limited only to neurons that are involved in a particular function, such as oviposition, the interactions between these neurons becomes more apparent. By applying RenEEL to a subconnectome consisting of the inputs to oviIN_R, we limited our analysis to finding partitions at a mesoscale and microscale level of relevance.

Generalized modularity (Eq. 1) offers an established method with which to probe mesoscale and microscale structure. The resolution parameter can be adjusted to retrieve more modules containing fewer neurons. A previous study demonstrated that maximizing generalized modularity reveals the hierarchically nested anatomical structure of the brain when applied to the full Hemibrain network (Kunin et al., 2023). The modules identified at higher resolutions were generally subsets of the coarsest modules, though this partitioning was not always strict. Even still, the high resolution modules of the full Hemibrain did not correspond to the coarse modules that we obtained for the subconnectome consisting of the inputs to oviIN_R (Fig. 5*B, C*). This demonstrates that maximizing classical (i.e., coarse) modularity on a subconnectome does not produce the same results as maximizing generalized modularity at higher resolutions on a full connectome. Although our study did not set out to investigate the subtleties between classical and generalized modularity analyses, we propose that applying classical modularity to a subconnectome targets a different scope of brain structure than applying generalized modularity on the full connectome. Future work is needed to gain a comprehensive understanding of how community-detection tools can be honed to reliably retrieve circuit structure in the *Drosophila* brain.

## Synthesis

Reviewing Editor: Mark Sheffield, University of Chicago Division of the Biological Sciences Decisions are customarily a result of the Reviewing Editor and the peer reviewers coming together and discussing their recommendations until a consensus is reached. When revisions are invited, a fact-based synthesis statement explaining their decision and outlining what is needed to prepare a revision will be listed below. The following reviewer(s) agreed to reveal their identity: Zepeng Yao. Note: If this manuscript was transferred from JNeurosci and a decision was made to accept the manuscript without peer review, a brief statement to this effect will instead be what is listed below.

This manuscript presents a well-executed and insightful connectomic analysis of the Drosophila oviposition circuit, focusing on inputs to the inhibitory neuron oviIN. Using the Hemibrain EM dataset and the RenEEL clustering algorithm, the authors identify oviIN as a major integrative hub and reveal that its inputs can be grouped into seven discrete modules, potentially representing parallel pathways for behavioral control. Both reviewers agree that this work makes a valuable contribution to understanding how sensory and internal signals may be integrated within a key behavioral circuit and highlights an exciting foundation for future functional studies.

The reviewers find the methods to be rigorous, the interpretations largely sound, and the manuscript well written. However, they suggest the manuscript could be further strengthened by modest expansions in scope and clarification of key details. One reviewer notes that the focus on direct inputs to oviIN-while methodologically motivated-limits broader conclusions about circuit-level structure, and raises the possibility that including "two-hop" connections or inputs to other key oviposition neurons (e.g., oviEN or oviDN) could yield deeper insights. Even a partial comparison or quantification of shared inputs across circuit nodes would enhance the study's generalizability.

Both reviewers encourage improvements to clarity and accessibility. Terminology like "strong input," "SNP," and "super neuropil" should be more clearly defined, and key figures-such as Figures 3, 4, 5, and related supplements-would benefit from improved labeling, readability, and alternative visualizations (e.g., replacing pie charts with bar graphs). Technical details, such as how colocalization or input strength thresholds were defined, should be explained earlier and more explicitly. The authors are also encouraged to ensure that the GitHub code repository is accessible and to verify the consistency of terms and formatting throughout.

Detailed reviewer comments follow below.

Reviewer 1:

This study uses the connectome to analyze inputs to the known oviposition circuit in Drosophila. The authors find that the inhibitory neuron in the circuit, oviIN, is a major hub within the circuit and more broadly, as it makes more connections and receives more direct inputs than almost any other neuron in the entire brain. The authors thus focus on inputs to oviIN and identify 7 clusters of input neurons, which may represent parallel circuits.

The results are generally convincing and provide new information regarding inputs to the oviposition circuit, a circuit that must integrate sensory and internal information to promote adaptive behavioral decisions.

I have a couple of general comments:

1) By focusing on a "subconnectome" that only includes direct inputs to oviIN, it's hard to know what the modules really represent. The authors explain why they do this (to avoid simply recapitulating the brain's overall neuropil structure), but that doesn't prove that their approach can pull out clusters that represent "circuits", as many circuits are likely to be defined by connections that are not included in the "subconnectome". For example, if the authors included neurons that are directly connected to oviIN inputs, or included "two-hop" connections between oviIN inputs, would the results change substantially?

2) I can understand why the authors focused on oviIN, but I think the paper would be more interesting if they also analyzed inputs to oviEN and oviDN. This would allow the authors to make a broader claim that they are analyzing inputs to the egg-laying circuit rather than just inputs to oviIN. Even if a full analysis is not possible, it might be interesting to know - for example - how many oviIN inputs are also connected to oviEN or oviDN, or vice versa.

Minor comments:

- I'm not able to access the Github page cited with the code for the paper (https://github.com/orgs/Gutierrez-lab/oviIN-inputs).

- Please explain how "typical" vs. "atypical" MBONs are defined.

- Please list what the abbreviations in Figure 5 and Figure 4 supplement 2 stand for (even if they are defined in a previous study). Also, I am not sure that "super neuropil" is a standard term; I didn't find it in the Ito paper.

- Some parts of figures are too small and hard to read, e.g., pie charts in Figure 5A, diagrams to the right of the Figure 5B graph, graphs in Figure 6B, labels in Figure 4 supplement 1.

- Pie charts are rarely the best way to visualize data, as the size of wedges can be misleading; clustered or stacked bar graphs would convey the same point more effectively.

Reviewer 2:

Drosophila oviposition, during which a female fruit fly integrates various sensory and contextual signals to select an egg-laying site, is an established model for studying complex decision making. The neural circuit for oviposition has been partially worked out recently. In this manuscript, Langstaff et al. use the Hemibrain EM connectome to examine the previously described circuit and identify the oviIN neurons as a potential hub for signal integration. The authors show that the oviINs receive tens of thousands synaptic connections from nearly 1,000 cell types, making them one of the neurons that receive the most synaptic inputs in the brain. Using a machine learning algorithm, RenEEL, the authors demonstrate that the oviIN presynaptic partners can be partitioned into seven discrete modules, each differing in their synaptic input areas onto the oviINs and their neuropil coverage. The authors propose that these modules may represent parallel pathways conveying different signals to the oviINs for oviposition decision making, laying a foundation for future functional characterization.

Although this study is primarily computational, it systemically characterizes the input neurons to the oviINs and sheds lights on their structural modularity, which will undoubtedly inspire future experimental work. Moreover, the approaches employed here can be adapted to analyze functional modularity in other neural circuits or brain regions. Therefore, I believe this study is significant, will be well received by the growing connectomics field and the broader neuroscience community, and is suitable for publication in eNeuro.

Overall, the methods are solid, the results are well presented and interpreted, and the manuscript is well written. All my comments and suggestions below are minor. I recommend this manuscript for publication in eNeuro.

Minor:

1. Figure 1C: I suggest shifting the top labels (SAG, pC1a, etc.) slightly to the right. I found myself looking at the wrong columns occasionally.

2. Figure 3A: In the figure legend, clearly state that "strong inputs" in the figure mean 100 or more synaptic connections.

3. Figure 3B: Very minor: change "oviINr" in the axis labels to "oviIN_R" for consistency with the text and other figures.

4. Lines 103-104: SNP (Superior Neuropils of the Protocerebrum) is not as commonly used as the individual neuropil names in the Drosophila literature. It would be helpful to cite Ito et al., 2014 here and provide a brief description that SNP includes SMP, SIP, SLP, and LH. (Such information is currently in the Discussion section.)

5. Line 106: The ISNs described in Jourjine et al., 2016 are located in the SEZ, not the SNP. You can add González Segarra et al., eLife 2023 to the citation.

6. Line 114: It might be good to put the citation for the Hemibrain dataset here again.

7. Somewhere in the manuscript (perhaps early in the Results section), it might be helpful to clarify that in this manuscript, "strong" (or "weak") connections refer to connections with a large (or small) number of synapses.

8. Lines 180-181: List the 14 neuropils here or somewhere else in the manuscript?

9. Line 190: "for the majority of cell types": what is the percentage?

10. Line 456: The SNP may not be as well characterized as some other brain regions, but describing it as "mysterious" may not be appropriate.

## Author Response

eNeuro reviews Synthesis of Reviews:

Computational Neuroscience Model Code Accessibility Comments for Author (Required):

Reviewers were not able to load/access the Github page cited in the paper where the code should be located (https://github.com/orgs/Gutierrez-lab/oviIN-inputs). please make sure the link is correct and the code is accessible.

Synthesis Statement for Author (Required):

This manuscript presents a well-executed and insightful connectomic analysis of the Drosophila oviposition circuit, focusing on inputs to the inhibitory neuron oviIN. Using the Hemibrain EM dataset and the RenEEL clustering algorithm, the authors identify oviIN as a major integrative hub and reveal that its inputs can be grouped into seven discrete modules, potentially representing parallel pathways for behavioral control. Both reviewers agree that this work makes a valuable contribution to understanding how sensory and internal signals may be integrated within a key behavioral circuit and highlights an exciting foundation for future functional studies.

The reviewers ﬁnd the methods to be rigorous, the interpretations largely sound, and the manuscript well written. However, they suggest the manuscript could be further strengthened by modest expansions in scope and clariﬁcation of key details. One reviewer notes that the focus on direct inputs to oviIN-while methodologically motivated-limits broader conclusions about circuit-level structure, and raises the possibility that including "two-hop" connections or inputs to other key oviposition neurons (e.g., oviEN or oviDN) could yield deeper insights. Even a partial comparison or quantiﬁcation of shared inputs across circuit nodes would enhance the study's generalizability.

Both reviewers encourage improvements to clarity and accessibility. Terminology like "strong input," "SNP," and "super neuropil" should be more clearly deﬁned, and key ﬁgures-such as Figures 3, 4, 5, and related supplements-would beneﬁt from improved labeling, readability, and alternative visualizations (e.g., replacing pie charts with bar graphs). Technical details, such as how colocalization or input strength thresholds were deﬁned, should be explained earlier and more explicitly. The authors are also encouraged to ensure that the GitHub code repository is accessible and to verify the consistency of terms and formatting throughout.

We thank the reviewers for the time and attention they put into providing helpful feedback. We have addressed each individual comment below to the best of our ability. Some of these minor comments prompted us to make substantial changes to the ﬁgures and text to improve the clarity of our manuscript. We describe those additional changes here: ● We redid some of our initial analyses using a connection weight threshold of 3 whereas we previously did not implement any threshold. Connection weights less than 3 are considered to be potentially erroneous by Scheﬀer et al (2020), and given that oviIN is a voluminous neuron, we thought that it might be more likely than smaller neurons to have erroneous synapses and we did not want that to distort the results. Our conclusions are unaﬀected by this change, but we felt that it was an important revision to make for the sake of instilling conﬁdence in the reader about our results. The rescaled y-axes in Figures 1B, 1D, and 3A reﬂect the redone analyses using the new connection weight threshold, as do the slightly reordered rankings in Figure 2. The quantities in the main text have also been updated to reﬂect the new synaptic partner and synapse weight counts after a connection weight threshold of 3 was implemented. We note that we did not implement a connection weight threshold for maximizing the modularity of the subconnectome of inputs to oviIN. That means that neurons that connect to oviIN with a weight of less than 3 were included in the subconnectome, as were the edges between subconnectome neurons that are less than 3. Our rationale for not applying a threshold for the modularity analysis was because we wanted to be overly inclusive in our subconnectome. The direct inputs to oviIN are unlikely to represent the entirety of the specialized circuits we sought, but rather fragments of circuits at best. Omitting edges from a network has serious implications for the algorithm. We felt that we would obtain the best results by including all connections, however small, to avoid isolating any nodes that could be potentially informative to the clustering algorithm. We now indicate which analyses use a connection weight threshold in the Results and Methods sections and in the ﬁgure captions where applicable. Our rationale for not using a connection threshold when maximizing modularity of the subconnectome is also now included in the Methods section. ● An Extended Data section was added to conform to the formatting requirements for eNeuro. Supplemental ﬁgures and tables were moved to that section. We have included additional ﬁgures in the Extended Data section that are motivated by the reviewer comments we address below. ● Figure 5A was previously a pie chart where the pie slices represented the proportion of connections made within each supercategory by all the neurons in a module, but normalized on a per neuron basis. For each neuron, the proportion of synapses made within each supercategory were calculated by totaling the synapse weights made by the neuron in that supercategory and dividing by the total synapse weights made by the neuron in all the supercategories. These proportions were tallied up by supercatgory for each module. This pie chart plot was replaced with a bar chart that is more intuitive. It simply shows the tally of input synapses to the neurons in each module by the supercategory in which those input synapses are located. ● Figure 6 has been reconﬁgured and revised. The matrix of histograms has now been moved to the Extended Data section and has been replaced with a heatmap showing the z-scores of the pooled similarity among pairs of modules. The two heatmaps in Figure 6 now complement each other as they provide information at a glance about the mean of the pooled similarity and the deviation from the random model. However, we maintained the matrix of histograms as an Extended Data ﬁgure since it contains a lot of raw information and some readers may prefer to view those instead of, or in addition to, the concise heatmaps in the main text. Additionally, the analysis in Figure 6 was redone with subconnectome neurons excluded from the inputs to the subconnectome neurons. Thus we analyzed the similarity of the subconnectome neurons on the basis of their non-subconnectome inputs. ● All ﬁgure captions were substantially revised to reﬂect the revised ﬁgures and for better clarity overall, even when no speciﬁc reviewer comments were given. ● The Methods section has been revised in keeping with the aforementioned changes to the analyses. ● The manuscript sections were reordered to conform to the formatting requirements of eNeuro. As such, some of the text from the Introduction was moved into the Discussion to adhere to the requirement that the Introduction be no more than 750 words.

Detailed reviewer comments follow below.

Reviewer 1:

This study uses the connectome to analyze inputs to the known oviposition circuit in Drosophila. The authors ﬁnd that the inhibitory neuron in the circuit, oviIN, is a major hub within the circuit and more broadly, as it makes more connections and receives more direct inputs than almost any other neuron in the entire brain. The authors thus focus on inputs to oviIN and identify 7 clusters of input neurons, which may represent parallel circuits.

The results are generally convincing and provide new information regarding inputs to the oviposition circuit, a circuit that must integrate sensory and internal information to promote adaptive behavioral decisions.

I have a couple of general comments:

1) By focusing on a "subconnectome" that only includes direct inputs to oviIN, it's hard to know what the modules really represent. The authors explain why they do this (to avoid simply recapitulating the brain's overall neuropil structure), but that doesn't prove that their approach can pull out clusters that represent "circuits", as many circuits are likely to be deﬁned by connections that are not included in the "subconnectome". For example, if the authors included neurons that are directly connected to oviIN inputs, or included "two-hop" connections between oviIN inputs, would the results change substantially? If we include 2-hop connections upstream of oviIN_R, we end up with a set of 18,374 unique neurons that are direct inputs to oviIN_R and direct inputs to those inputs. This is nearly the same amount of traced, non-cropped neurons in the full Hemibrain data (21,739 unique neurons). For that reason, we only performed the modularity maximization on the subconnectome that contained the 1,832 unique, traced, non-cropped neurons that are direct inputs (1-hop) to oviIN_R. A previous study performed modularity maximization on the full Hemibrain data. We compare those results to the modularity results for the full Hemibrain from Kuni n et al . (2023) in our Figures 5B &5C.

Your comment helped us to realize that we need to be more careful in how we interpret and discuss the modularity results. We cannot claim that modularity maximization forms clusters that deﬁnitively represent circuits in the brain. Our approach merely forms clusters that have denser intra-connectedness than inter-connectedness. Two neurons that are connected to each other are more likely to be in the same circuit than two neurons that are not connected, but there is no guarantee that a pair of connected neurons belongs to the same circuit, nor that two unconnected neurons don't belong to the same circuit. We used modularity maximization to get a ﬁrst approximation of the organization inherent in the abundant inputs to oviIN. Being able to associate uncharacterized cell types with each other is a far cry from discovering novel circuits, but it is a ﬁrst step. Most importantly, this approach enabled us to determine to what extent the primary strong inputs are independent "spokes" of a hub. Instead of the primary strong inputs coalescing into a single module, we found that there are primary strong inputs in every module. This suggests that there are parallel streams of inputs converging onto the oviIN. Ultimately, our approach revealed some approximate organization among oviIN's inputs that may someday support the discovery of the circuits that are involved in the integration of sensory information and the computations of evaluation and decision-making in oviposition. We have revised the Discussion section to more carefully state what our approach does and does not achieve. The above points are articulated most directly in the sub-section titled "High-level processing in the SNP" in the Discussion. We have also edited the Abstract, the Signiﬁcance Statement, the Introduction, and the Results to ensure that we don't suggest having identiﬁed novel circuits in our current study.

2) I can understand why the authors focused on oviIN, but I think the paper would be more interesting if they also analyzed inputs to oviEN and oviDN. This would allow the authors to make a broader claim that they are analyzing inputs to the egg-laying circuit rather than just inputs to oviIN. Even if a full analysis is not possible, it might be interesting to know - for example - how many oviIN inputs are also connected to oviEN or oviDN, or vice versa.

We are currently analyzing the inputs to oviEN and writing a separate manuscript. Including those analyses here would make the current manuscript unwieldy. However, we agree that some amount of comparison between oviIN and oviEN would be worthwhile to include in our submitted manuscript.

If we only consider connections with a weight of 3 or greater, we ﬁnd that oviIN_R and oviEN_R have 37 presynaptic partners in common, which is a small portion of the presynaptic partners to oviEN_R (235) and to oviIN_R (1180). Only 3 of those 37 overlapping partners are cell types that make strong inputs to oviIN_R (i.e. the cell type makes more than 100 synaptic connections to oviIN_R). Among those, only the SMP551 is a primary strong input to oviIN (meaning that it primarily targets oviIN) and an overlapping input to oviEN. We ﬁnd a similar level of overlap between oviIN_R and the combined right hemisphere instances of the 3 oviDN subtypes (oviDNa, oviDNb, and SLP410).

The lack of appreciable overlap in inputs justiﬁes separate investigations of oviIN and oviEN, but is also worth noting explicitly in the present manuscript. We have incorporated a description of the overlap in pre-synaptic inputs to the oviIN, oviEN, and oviDNs in the ﬁrst section, third paragraph of the Results. The Venn diagram below has now been included as Extended Data Figure 1-1, and it is cited halfway through the third paragraph of the Results. We have also made it clear in the writing that we are not analyzing inputs to the full oviposition circuit, but rather to the oviIN, which gets an unexpectedly large amount of the inputs to the circuit.

Minor comments:

- I'm not able to access the Github page cited with the code for the paper (https://github.com/orgs/Gutierrez-lab/oviIN-inputs).

Apologies for the oversight. The repository permissions had been set to private but we have now made our Github repository public and updated the link. Our public repository can now be accessed here: https://gi thub.com/Guti errez-l ab/ovi IN-i nputs - Please explain how "typical" vs. "atypical" MBONs are deﬁned.

Atypical MBONs are mushroom body output neurons that receive inputs from areas outside of the mushroom body, as determined by Li et al ., 2020. These MBONs are thought to integrate other types of sensory information together with the Kenyon Cell inputs they receive. Atypical MBONs are now brieﬂy deﬁned in the 5th paragraph of the Results subsection titled "The modules divide the primary strong inputs to oviIN_R" where they are ﬁrst mentioned.

- Please list what the abbreviations in Figure 5 and Figure 4 supplement 2 stand for (even if they are deﬁned in a previous study). Also, I am not sure that "super neuropil" is a standard term; I didn't ﬁnd it in the Ito paper.

Thank you for pointing out the confusion in our abbreviations and terminology. "Super neuropil" is not a standard term, thus we have replaced it with the accepted term, "Supercategory", as deﬁned by Ito et al ., 2014. The term has been corrected throughout the paper, speciﬁcally in the captions for Figure 5A and Extended Data Figure 4-2. To further clarify the relationship between supercategories and neuropils, and to deﬁne the abbreviations used in our Figures, we now include a supplementary ﬁgure that lists the neuropils and their abbreviations, their full names, and their supercategories. This ﬁgure is now included as Extended Data Figure 5-1.

- Some parts of ﬁgures are too small and hard to read, e.g., pie charts in Figure 5A, diagrams to the right of the Figure 5B graph, graphs in Figure 6B, labels in Figure 4 supplement 1.

We have modiﬁed the layout of Figure 6 so that it is more readable. Figure 6 now has two panels with heatmaps for the mean pooled input similarity (Fig. 6A) and the z-score of the pooled input similarity (Fig. 6B). The upper triangular matrix of histograms for the input similarities has been moved to the Extended Data section as a supplement to Figure 6.

We have also increased the size of text in Figure 5 and the size of the neuropil diagrams on the right of Figure 5B. The font sizing in all ﬁgures has been made more consistent to ensure legibility throughout.

- Pie charts are rarely the best way to visualize data, as the size of wedges can be misleading; clustered or stacked bar graphs would convey the same point more eﬀectively.

We have taken your suggestion and switched to stacked bar charts to visualize the neuropil composition of each module in Figure 5A and the neuropils in which inputs are made to oviIN_R in our Extended Data Figure 4-2. These new ﬁgures now provide more information than the pie charts previously, and they are more intuitive. Each ﬁgure now shows the synapse counts along the y-axis by supercategory for each module along the x-axis. For Figure 5A, the synapse counts are the total counts of input synapses to the neurons in a given module. For Extended Data Figure 4-2, the synapse counts are the input synapse counts to oviIN_R from a given module.

Reviewer 2:

Drosophila oviposition, during which a female fruit ﬂy integrates various sensory and contextual signals to select an egg-laying site, is an established model for studying complex decision making. The neural circuit for oviposition has been partially worked out recently. In this manuscript, Langstaﬀ et al. use the Hemibrain EM connectome to examine the previously described circuit and identify the oviIN neurons as a potential hub for signal integration. The authors show that the oviINs receive tens of thousands synaptic connections from nearly 1,000 cell types, making them one of the neurons that receive the most synaptic inputs in the brain. Using a machine learning algorithm, RenEEL, the authors demonstrate that the oviIN presynaptic partners can be partitioned into seven discrete modules, each diﬀering in their synaptic input areas onto the oviINs and their neuropil coverage. The authors propose that these modules may represent parallel pathways conveying diﬀerent signals to the oviINs for oviposition decision making, laying a foundation for future functional characterization.

Although this study is primarily computational, it systemically characterizes the input neurons to the oviINs and sheds lights on their structural modularity, which will undoubtedly inspire future experimental work.

Moreover, the approaches employed here can be adapted to analyze functional modularity in other neural circuits or brain regions. Therefore, I believe this study is signiﬁcant, will be well received by the growing connectomics ﬁeld and the broader neuroscience community, and is suitable for publication in eNeuro.

Overall, the methods are solid, the results are well presented and interpreted, and the manuscript is well written. All my comments and suggestions below are minor. I recommend this manuscript for publication in eNeuro.

Minor:

1. Figure 1C: I suggest shifting the top labels (SAG, pC1a, etc.) slightly to the right. I found myself looking at the wrong columns occasionally.

The labels in Figure 1C have been reconﬁgured for better clarity. In addition, the labels in Figures 1B and 1D have been shifted for improved interpretability.

2. Figure 3A: In the ﬁgure legend, clearly state that "strong inputs" in the ﬁgure mean 100 or more synaptic connections.

The caption for Figure 3A has been updated to clearly state that strong inputs are cell types that make 100 or more synaptic connections to oviIN and that this is what is shown in the dark purple parts of the ﬁgure. Likewise, weaker inputs are cell types making less than 100 synaptic connections to oviIN and are shown in light purple.

3. Figure 3B: Very minor: change "oviINr" in the axis labels to "oviIN_R" for consistency with the text and other ﬁgures.

That change has been made in Figure 3B and in all other ﬁgures so that "oviINr" is replaced by "oviIN_R". We have also gone through the main text and replaced all instances of "oviINr". We use "oviIN_R" when referring to the right hemisphere instance of the oviIN, and we use "oviIN" when referring to the cell type generally.

4. Lines 103-104: SNP (Superior Neuropils of the Protocerebrum) is not as commonly used as the individual neuropil names in the Drosophila literature. It would be helpful to cite Ito et al., 2014 here and provide a brief description that SNP includes SMP, SIP, SLP, and LH. (Such information is currently in the Discussion section.) We agree with your comment and ﬁnd it to be important to provide more description of the SNP, not only the neuropils that compose it, but also the historical understanding of it as a "diﬀuse" set of neuropils without obvious function that is revealed by its structure (or lack thereof). We have provided more information in this part of the Introduction about the SNP as suggested (last paragraph of Introduction). A citation for Ito et al . (2014) has been included here as well.

5. Line 106: The ISNs described in Jourjine et al., 2016 are located in the SEZ, not the SNP. You can add González Segarra et al., eLife 2023 to the citation.

Thank you for noticing this oversight. The ISNs described in Jourj i ne et al . (2016) are indeed located in the SEZ; however, their downstream partners were shown to project to the SMP by Gonzal ez-Segarra et al . (2023) . The Janu-AstA neurons found by Landayan et al . (2021) also project to the SMP and similarly have a role in regulating water and food intake. For this part of the Introduction, our goal is to point out that there are circuits involved in internal states and higher order processing in the SNP. We have revised these sentences in the last paragraph of the Introduction to more clearly indicate what is known about the circuits that include projections in the SNP while maintaining brevity since we return to this topic in the Discussion subsection titled "High-level processing in the SNP".

González Segarra, A.J., Pontes, G., Jourjine, N., Del Toro, A., and Scott, K. (2023). Hunger- and thirst-sensing neurons modulate a neuroendocrine network to coordinate sugar and water ingestion. eLife 12, RP88143. https://doi.org/10.7554/eLife.88143..

Jourjine, N., Mullaney, B.C., Mann, K., and Scott, K. (2016). Coupled Sensing of Hunger and Thirst Signals Balances Sugar and Water Consumption. Cell 166, 855-866. https://doi.org/10.1016/j.cell.2016.06.046.

Landayan D, Wang BP, Zhou J, Wolf FW Thirst interneurons that promote water seeking and limit feeding behavior in Drosophila. eLife, 10:e66286. hps://doi.org/10.7554/eLife.66286 6. Line 114: It might be good to put the citation for the Hemibrain dataset here again.

We have included the citation for Scheﬀer et al (2020) in that opening sentence of the Results, as suggested.

7. Somewhere in the manuscript (perhaps early in the Results section), it might be helpful to clarify that in this manuscript, "strong" (or "weak") connections refer to connections with a large (or small) number of synapses.

Thanks for the suggestion. We now deﬁne what we consider a strong connection among cell types in the third paragraph in the ﬁrst section of the Results. We deﬁne strong synaptic connections among cell types as >= 100 connection weights. In other places in the text where we make a more qualitative comparison or description of the strength of a connection, for example, between individual neurons rather than cell types, we are careful to make those observations in more relative terms to avoid confusion.

8. Lines 180-181: List the 14 neuropils here or somewhere else in the manuscript? We created a supplemental ﬁgure which contains all the Hemibrain neuropil abbreviations, full neuropil names, and supercategories from Ito et al ., 2014.

This ﬁgure is now included as Extended Data Figure 5-1. We initially refer to it in the "Parsing neuropil data" subsection of the Methods. It is also cited in the ﬁnal sentence of the "oviIN is an outlier in the brain" subsection in the Results, where we state that the oviIN_R makes connections within 5 supercategories. The reader is able to refer to the color-coded rows of Extended Data Figure 5-1 to see that oviIN_R connects with 14 out of the 63 Hemibrain primary neuropils. This ﬁgure is a supplement to Figure 5A to aid in identifying the neuropils from the abbreviations in the bar plot legends (as well as in Extended Data Figure 4-2).

9. Line 190: "for the majority of cell types": what is the percentage? The percentage is 56.25%, meaning that 56.25% of cell types that make strong inputs onto oviIN_R (>=100 synapses) have oviIN_R as their primary output. This statistic has now been included in the main text in the ﬁrst paragraph of the Results subsection titled "Hub-and-bespoke" structure among inputs to oviIN".

10. Line 456: The SNP may not be as well characterized as some other brain regions, but describing it as "mysterious" may not be appropriate.

We understand the concern and we have replaced "mysterious" with "understudied" in the ﬁrst sentence of the subsection titled "High-level processing in the SNP" in the Discussion.
