# Supplementary figures and images for: The Oviposition Inhibitory Neuron is a Potential Hub of Multi-Circuit Integration in the Drosophila Brain
Source: eNeuro. 2025 Sep 10;12(9):ENEURO.0123-25.2025. doi: 10.1523/ENEURO.0123-25.2025 (PMC12447867; doi:10.1523/ENEURO.0123-25.2025)

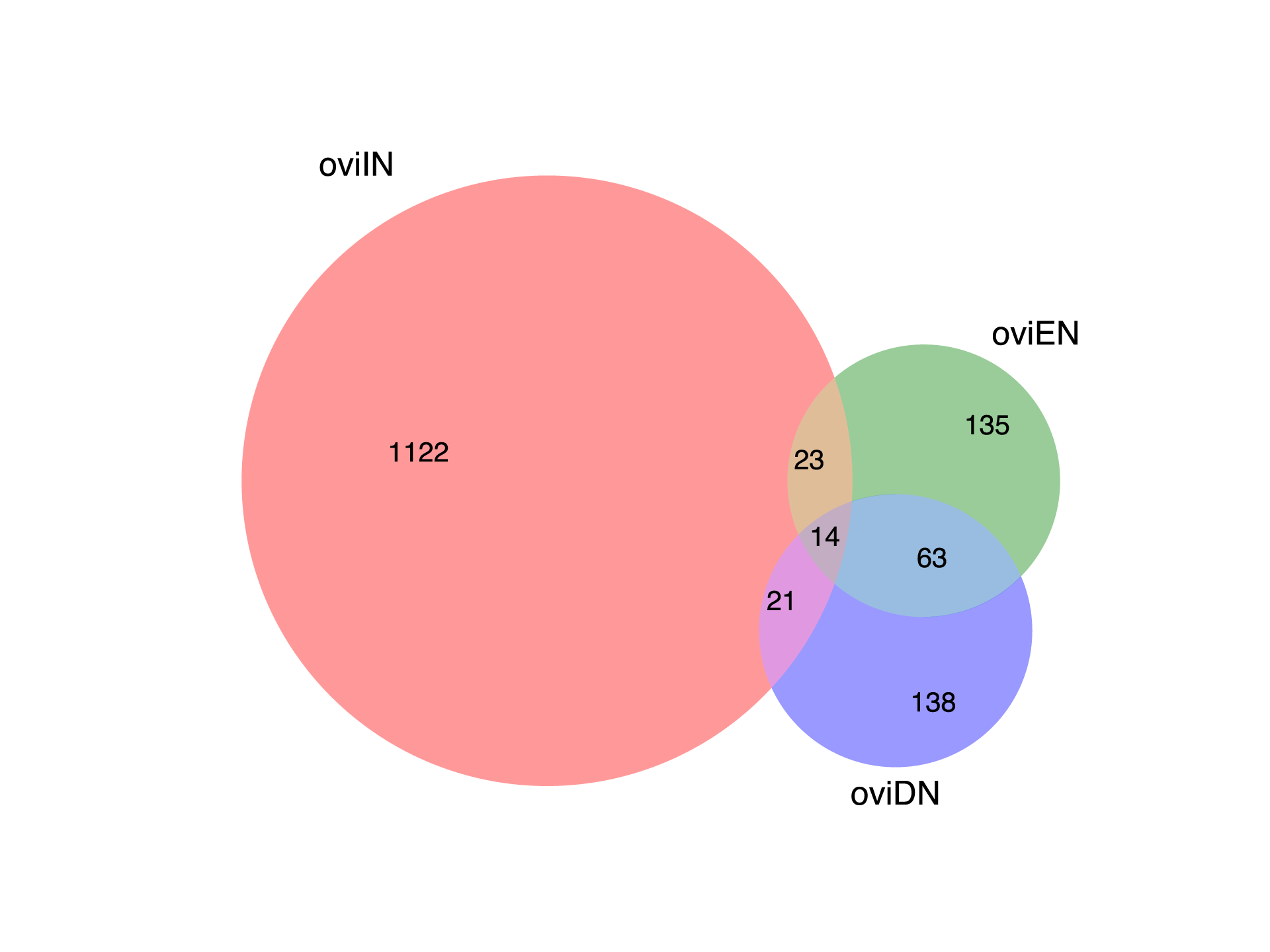

Supplement: Figure 1-1 — The overlap in unique presynaptic partners to oviIN_R (pink), SMP550_R (oviEN; green), and the combined group of right oviDN subtypes (oviDNa_R, oviDNb_R, and both instances of SLP410_R; purple). There are 14 unique neurons that are presynaptic to all 3 cell types. Download Figure 1-1, TIF file. [file eneuro-12-ENEURO.0123-25.2025-s009.tif]

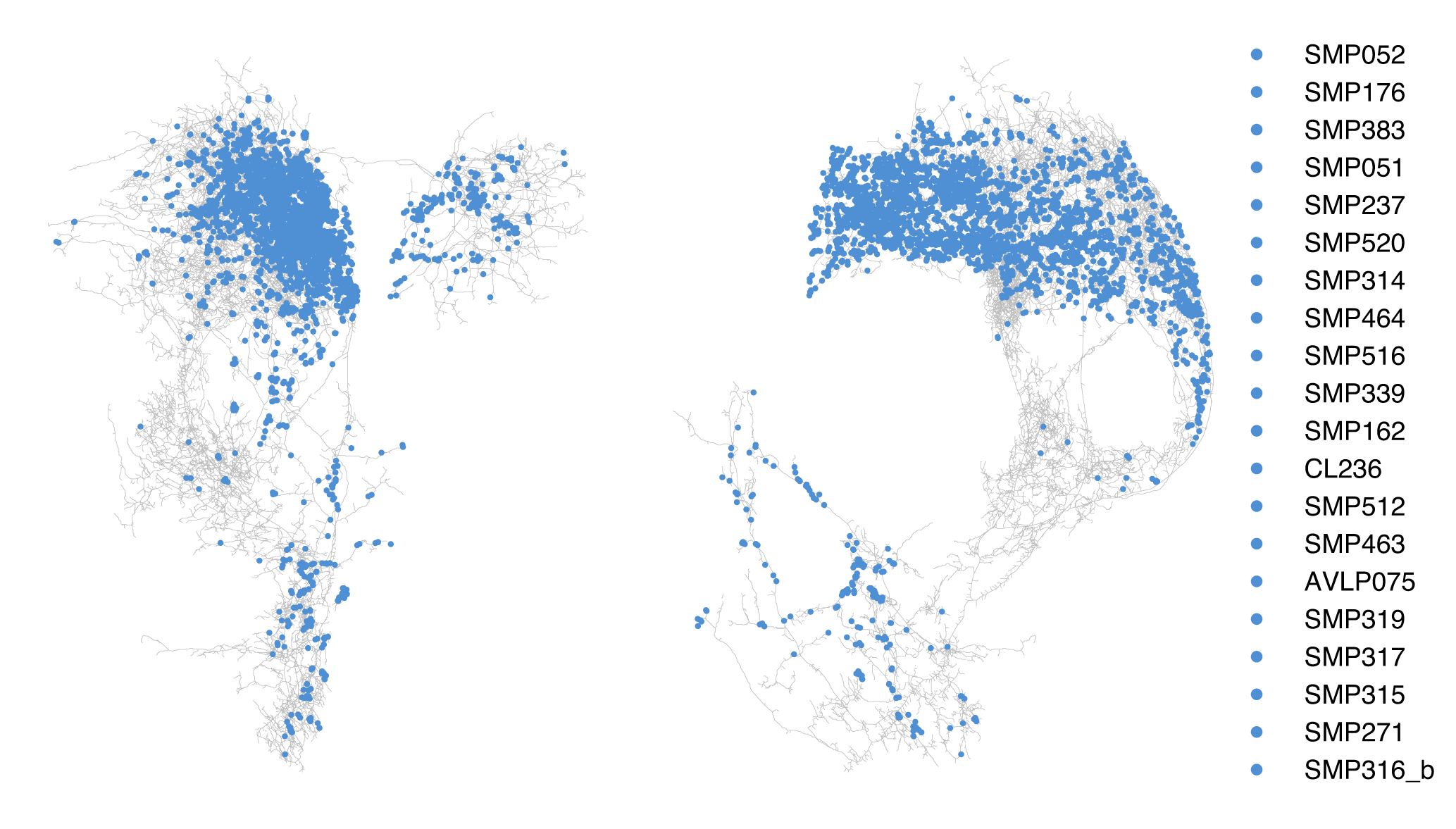

Supplement: Figure 4-1 — Synaptic input sites on oviIN_R from module 1. Left panel: front view; Right panel: lateral view. The 20 cell types from module 1 making the strongest connections to oviIN_R are shown in the legend. Download Figure 4-1, TIF file. [file eneuro-12-ENEURO.0123-25.2025-s010.tif]

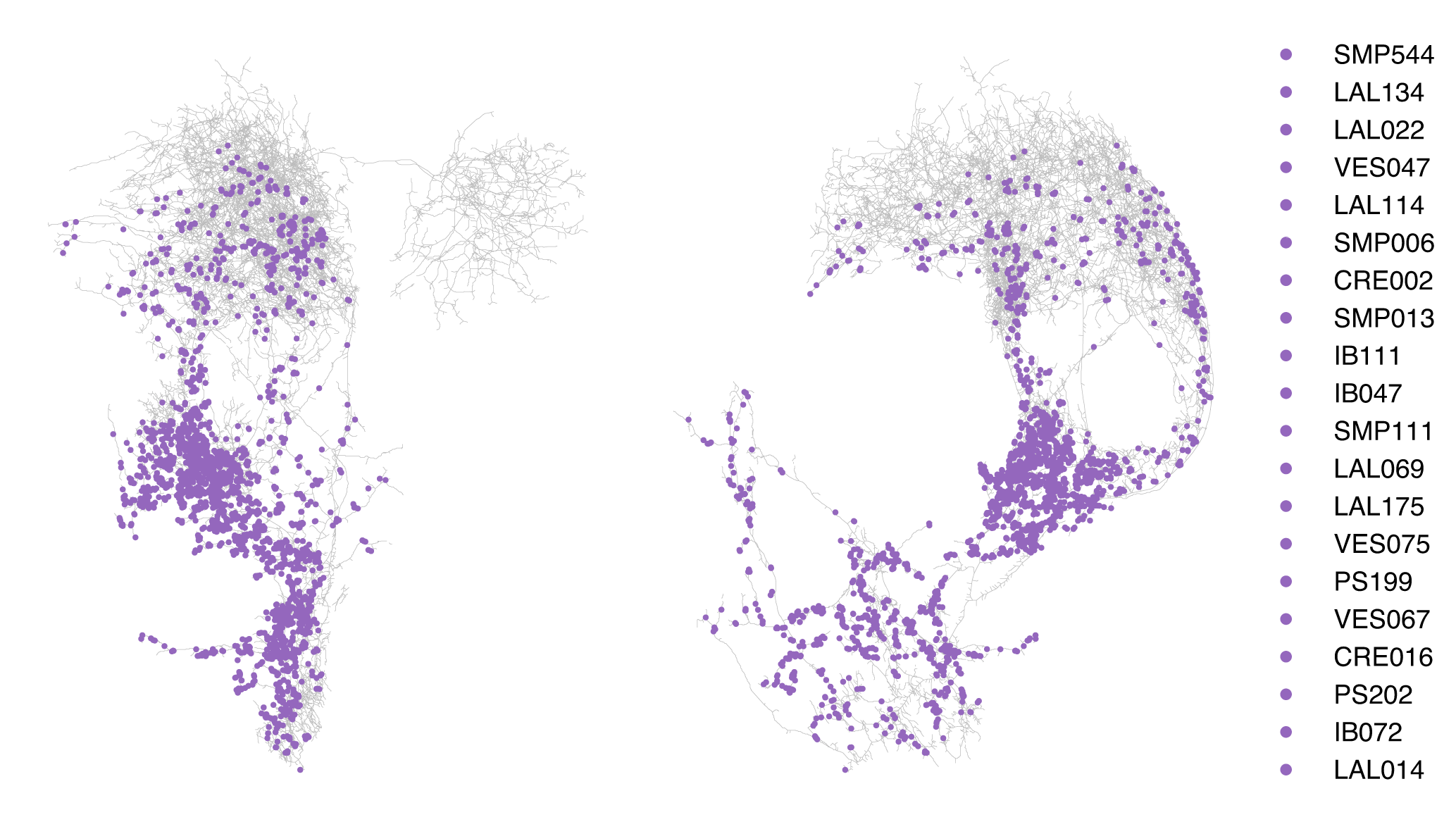

Supplement: Figure 4-2 — Synaptic input sites on oviIN_R from module 2. Left panel: front view; Right panel: lateral view. The 20 cell types from module 2 making the strongest connections to oviIN_R are shown in the legend. Download Figure 4-2, TIF file. [file eneuro-12-ENEURO.0123-25.2025-s011.tif]

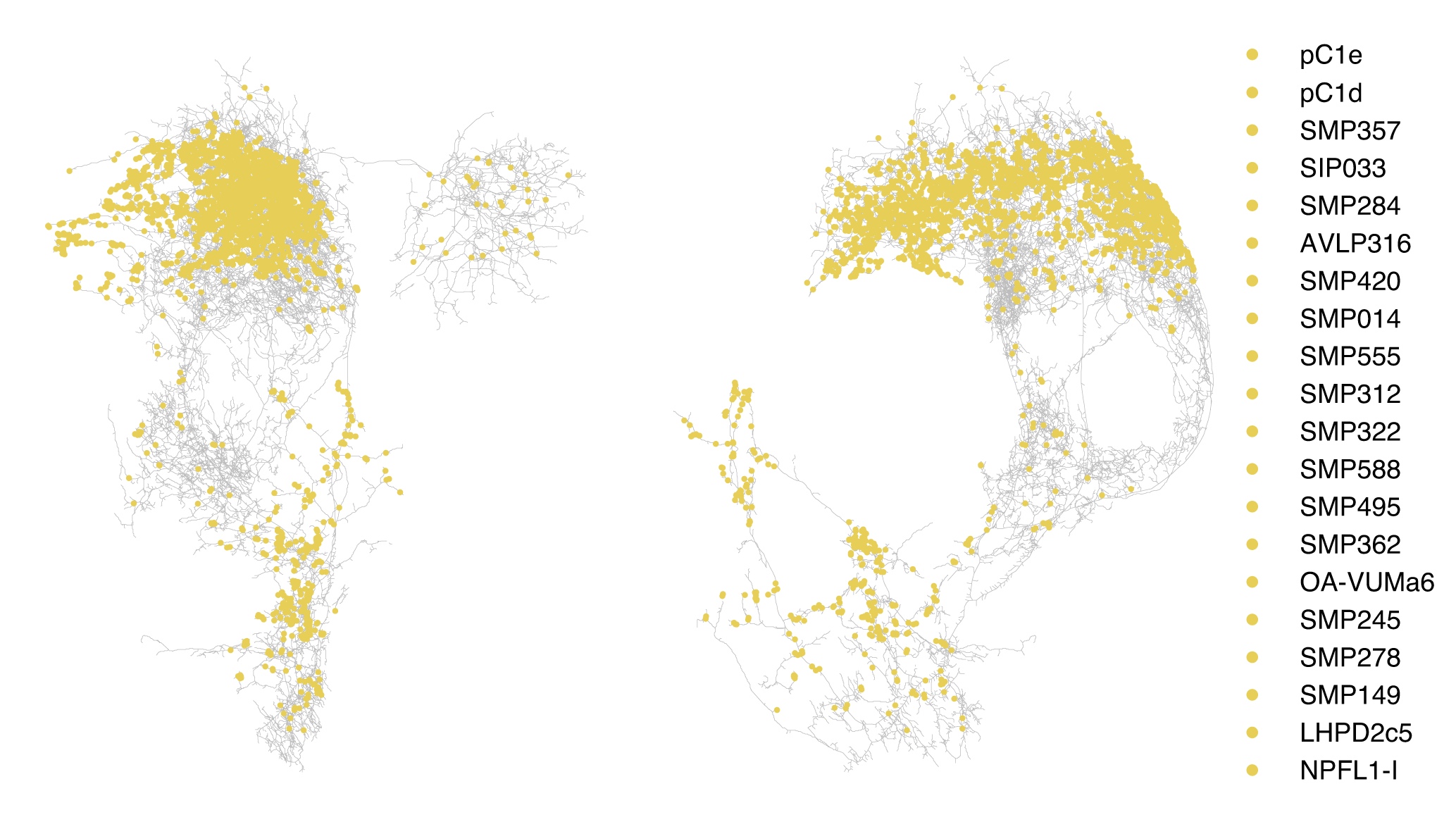

Supplement: Figure 4-3 — Synaptic input sites on oviIN_R from module 3. Left panel: front view; Right panel: lateral view. The 20 cell types from module 3 making the strongest connections to oviIN_R are shown in the legend. Download Figure 4-3, TIF file. [file eneuro-12-ENEURO.0123-25.2025-s012.tif]

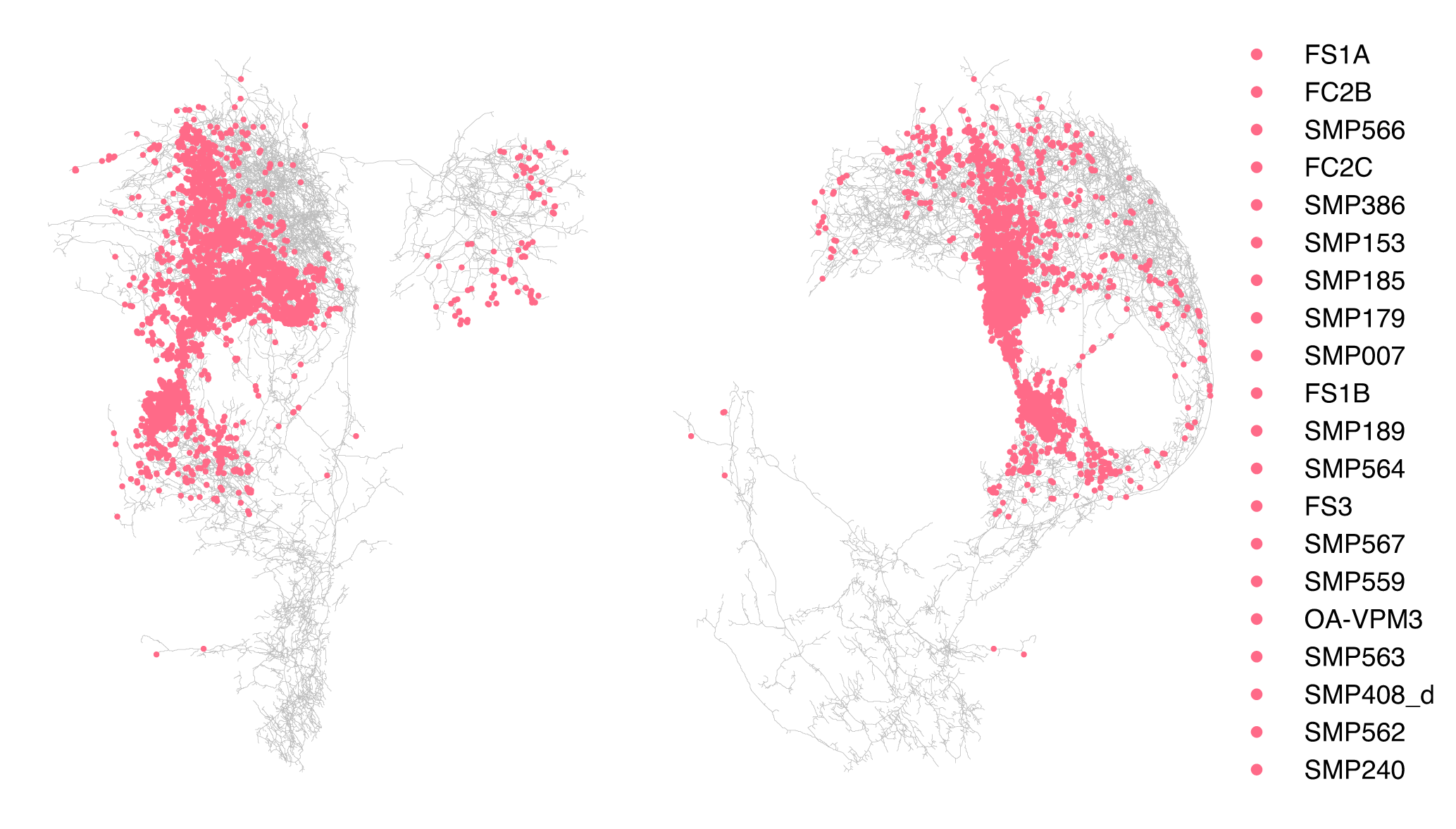

Supplement: Figure 4-4 — Synaptic input sites on oviIN_R from module 4. Left panel: front view; Right panel: lateral view. The 20 cell types from module 4 making the strongest connections to oviIN_R are shown in the legend. Download Figure 4-4, TIF file. [file eneuro-12-ENEURO.0123-25.2025-s013.tif]

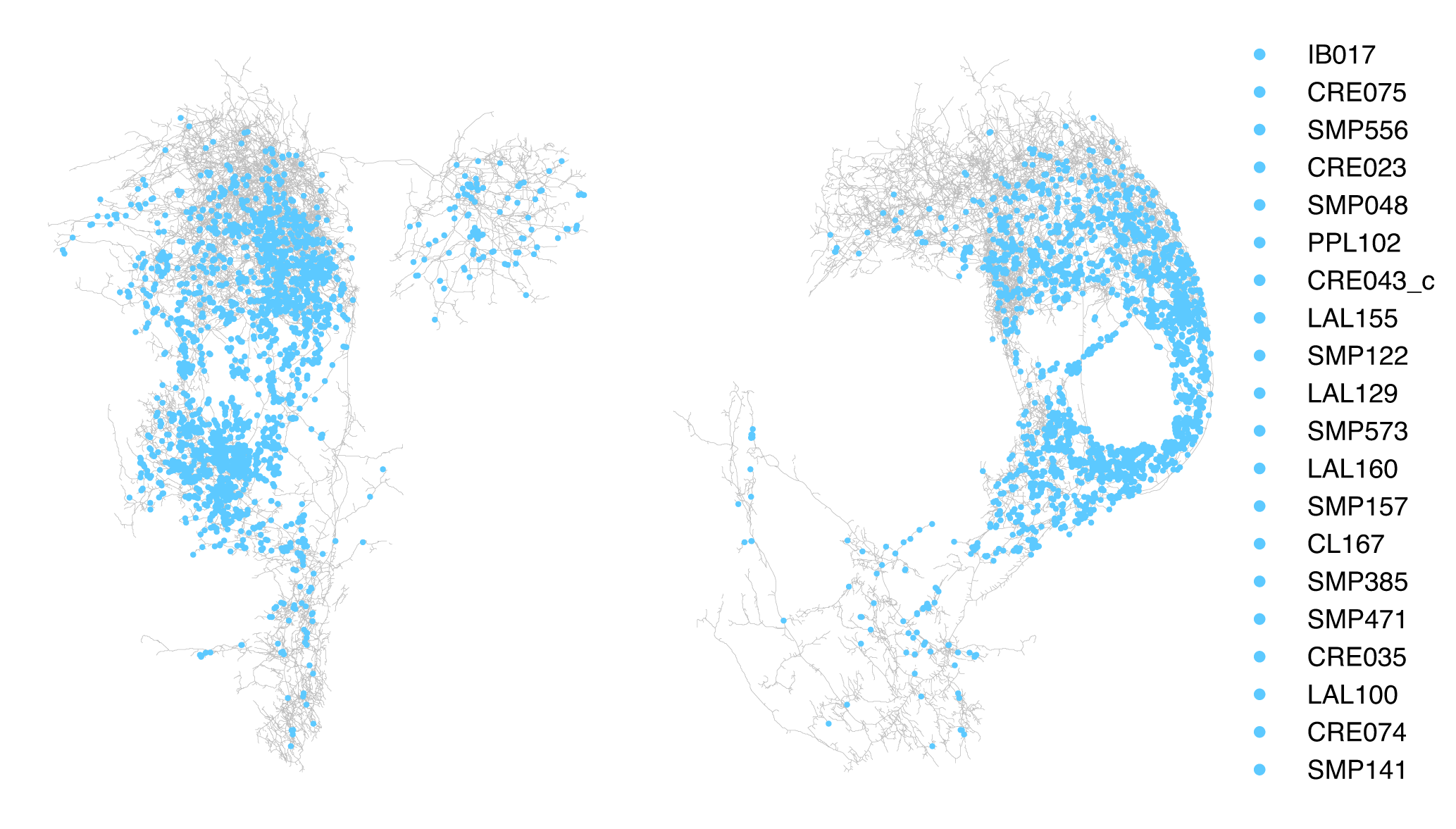

Supplement: Figure 4-5 — Synaptic input sites on oviIN_R from module 5. Left panel: front view; Right panel: lateral view. The 20 cell types from module 5 making the strongest connections to oviIN_R are shown in the legend. Download Figure 4-5, TIF file. [file eneuro-12-ENEURO.0123-25.2025-s014.tif]

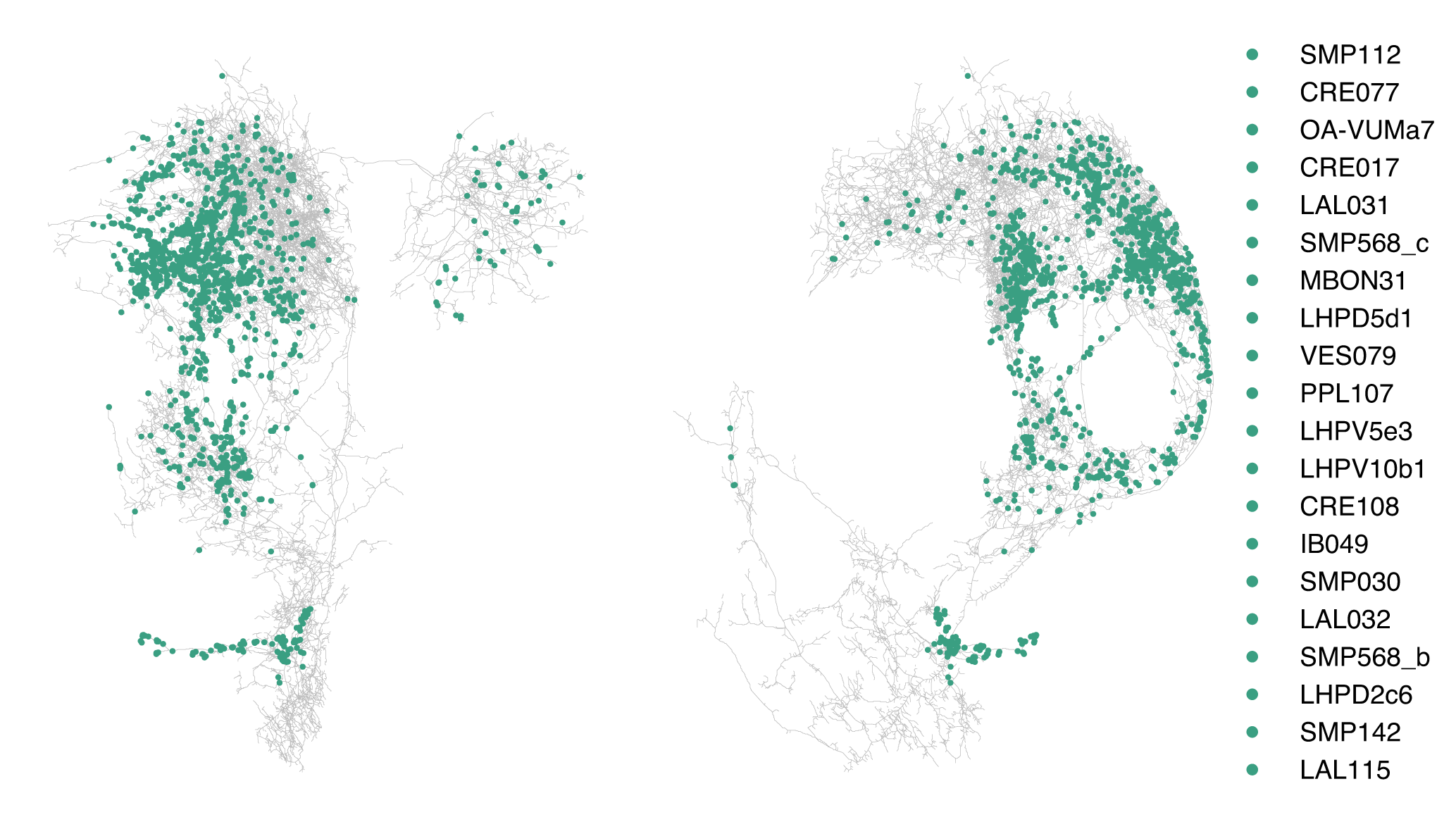

Supplement: Figure 4-6 — Synaptic input sites on oviIN_R from module 6. Left panel: front view; Right panel: lateral view. The 20 cell types from module 6 making the strongest connections to oviIN_R are shown in the legend. Download Figure 4-6, TIF file. [file eneuro-12-ENEURO.0123-25.2025-s015.tif]

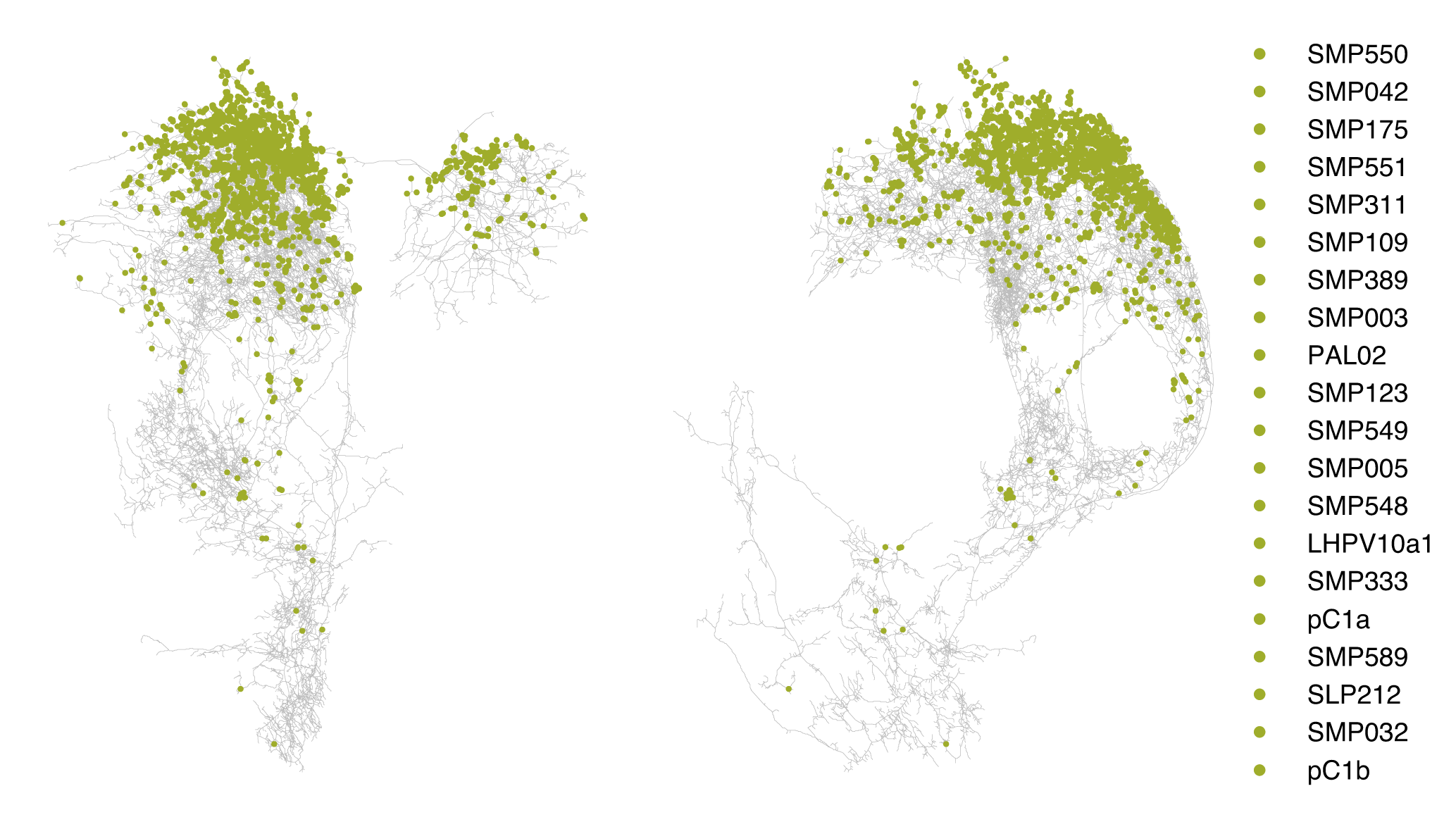

Supplement: Figure 4-7 — Synaptic input sites on oviIN_R from module 7. Left panel: front view; Right panel: lateral view. The 20 cell types from module 7 making the strongest connections to oviIN_R are shown in the legend. Download Figure 4-7, TIF file. [file eneuro-12-ENEURO.0123-25.2025-s016.tif]

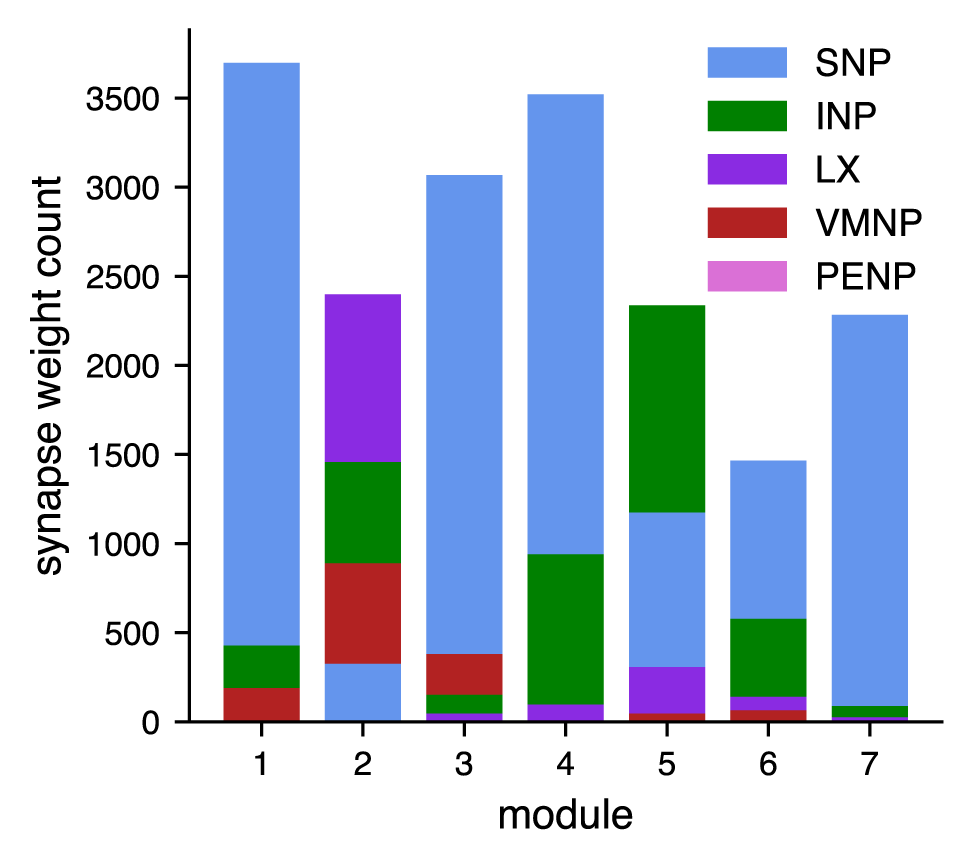

Supplement: Figure 4-8 — Supercategories where synapses from presynaptic partners to oviIN_R are localized. For the neurons within each module, the counts of synapses to oviIN_R within a neuropil are used to compute the supercategory synapse weight counts (see Figure 5-1). Download Figure 4-8, TIF file. [file eneuro-12-ENEURO.0123-25.2025-s017.tif]

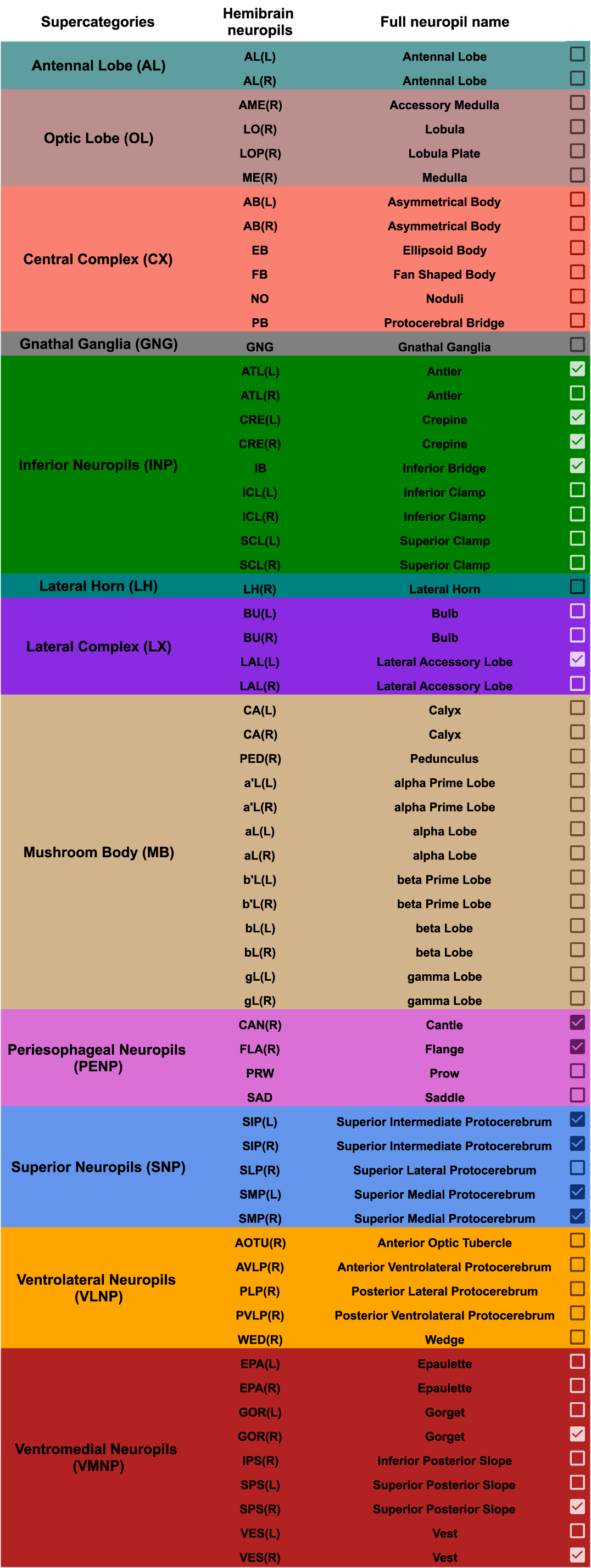

Supplement: Figure 5-1 — All Hemibrain neuropils from the v1.2.1 dataset along with their supercategories as described by Ito et al. (2014). The colors of each row correspond to the supercategory colors used in Figure 5A and Figure 4-8. A check mark in the last column denotes a neuropil where the oviIN_R receives connections. Download Figure 5-1, TIF file. [file eneuro-12-ENEURO.0123-25.2025-s019.tif]

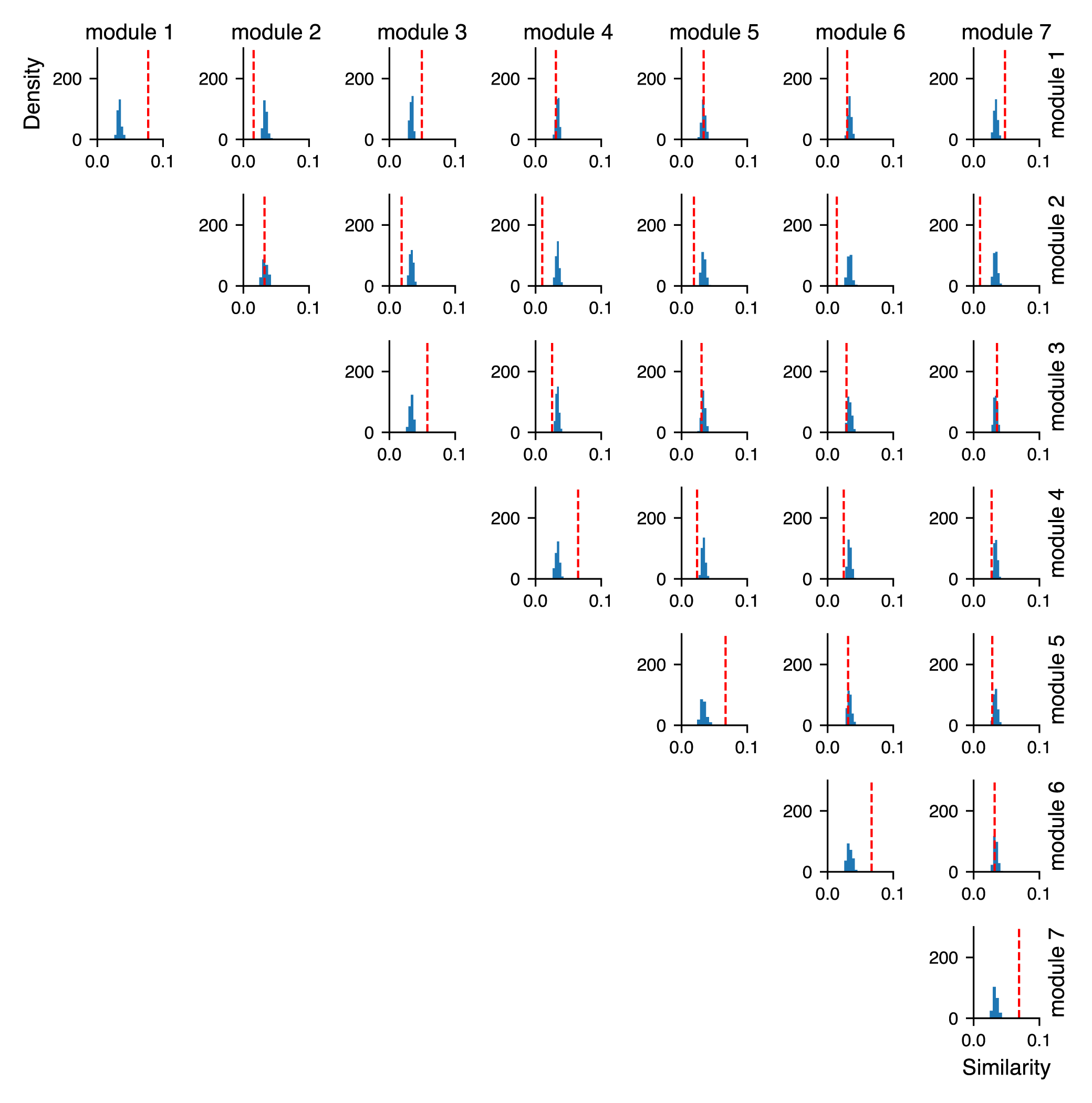

Supplement: Figure 6-1 — Randomization of module labels (n = 100 shuffles) shows decreased similarity for self-same cluster pairs. Input similarity is shown on the x-axes (similarity ranges from 0 to 1). The counts of similarity values are shown on the y-axes. The red dashed line in each plot indicates the mean input similarity between modules as visualized in Figure 6A. The histograms show the similarity scores for the random models. Along the diagonal, the mean input similarity among neurons in the same module (red dashed line) tends to be higher than the mean input similarity among neurons from random modules. In off-diagonal plots, the mean input similarity between neurons from two different modules (red dashed line) tends to be within the similarity distribution for neurons from random modules or lower than the distribution mean. Download Figure 6-1, TIF file. [file eneuro-12-ENEURO.0123-25.2025-s020.tif]
